# Supplementary material for: Patient and Family-Centered I-PASS SCORE Program: Resident and Advanced Care Provider Training Materials
Source: MedEdPORTAL. 2022 Aug 9;18:11267. doi: 10.15766/mep_2374-8265.11267 (PMC9360201; doi:10.15766/mep_2374-8265.11267)
Supplement: Supplementary file 1 — PFC I-PASS Core Content Module.m4vHealth Literacy Exercise.docxCore Content Module Evaluation.docxResident and Advanced Care Provider Workshop.pptxResident and Advanced Care Provider Facilitator Guide.docxPFC I-PASS Structure Role-Play.docxPFC I-PASS Rounds Role-Play.docxWritten Handoff for Rounds Role-Play.docxObserver Tool for Rounds Role-Play.docxRounds Report Simulation Activity.docxWorkshop Evaluation.docxBad Example of PFCR.mp4Good Example of PFCR.mp4Tips for Conducting Virtual Teaching Sessions.docx [file mep_2374-8265.11267-s001.zip › B. Health Literacy Exercise.docx]

**Health Literacy Exercise**

**Instructions:**

**Part 1:** You are taking care of Tom Sawyer (TS), just admitted with suspected Kawasaki disease (KD). His mother was with him on admission and now his father has arrived – the father is asking you to give an update and explain what happened. You have the history of present illness (HPI) – please convert it into a more health literate update for the father.

Remember:

- Use plain “living-room” language, avoiding medical jargon
- Organize information into 2-3 key concepts (“chunk”)
- Incorporate teach-back to demonstrate shared understanding (“check”)

**Formal History of Present Illness (HPI):**

TS is a 2 year old boy who presents to the primary care clinic with a history of rash, neck swelling, and fever for the past four days. History reveals that he has had double quotidian fever pattern over the past four days. He has been irritable and had a rash that is particularly prominent in the groin and on the trunk. His mother noted a swelling on the right side of his neck 3 days ago. She also noted erythema of both eyes for the past 4 days. In addition, she reports that he has had erythematous, dry, fissured lips, an erythematous tongue with a white exudate, and puffiness on the dorsal aspect of his hands and feet. On examination, the patient had all of the cardinal features of Kawasaki disease (KD), including a 2x3 centimeter enlarged lymph node in the right anterior cervical chain, an erythematous maculo-papular rash involving the trunk and upper extremities, swelling on the dorsal aspects of hands and feet, fissured erythematous lips, and a white strawberry tongue. Laboratory values include a leukocytosis, thrombocytosis, and an elevated erythrocyte sedimentation rate. He is admitted with a presumptive diagnosis of KD and will receive Intravenous Immunoglobulin (IVIG) this evening.

**Update for Father:**

**Part 2:** You are now preparing for family centered rounds the next day and developing the patient summary – please use the HPI on the previous page and the overnight updates to draft a health literate patient summary for rounds, focusing on Tom’s three problems as listed on the worksheet.

**Night Float Resident Report**

Tom completed his IVIG last night and continues to receive high-dose aspirin four times a day. He had a fever overnight, which came down with one dose of acetaminophen. The team was called twice for abdominal pain, but found Tom’s abdomen to be soft, non-tender, and non-distended. They considered obtaining an abdominal X-ray (KUB) but opted to continue to monitor and reevaluate in the morning.

**Nurse & Mother Overnight Report**

Both Tom’s mother and nurse report that Tom seems less cranky this morning, although he was complaining of a bellyache throughout the night. Tom’s mother reports that there were lots of tears when he cried. He has not had any diarrhea or vomiting. He has had poor oral intake, drinking just sips of Pedialyte from his sippy cup and refusing dinner and breakfast. Tom’s mother thinks his rash and cracked lips are improving.

**Pre-Rounding Data**

**Vital Signs:**

Tmax 39.5^o^C; Tc 37^o^C; HR 80-130bpm; RR 22-30 breaths/min; BP 74/45-110/60 mmHg; Pox 98-100% in room air

Input= 1050cc (maintenance IV fluids); Output = 500cc = 1.5 cc/kg/hr

**Physical Exam:**

GEN: sleeping comfortably in bed

HEENT: normocephalic, atraumatic; conjunctivae without hyperemia or discharge; no scleral icterus; nares patent; lips red without cracking; mucous membranes moist; drool noted on chin

Cardiac: normal S1S2, regular rate and rhythm, no murmur, rub, gallop

Lungs: clear to auscultation bilaterally, no wheeze, rales, rhonchi

Abd: non-distended, + bowel sounds, soft, non-tender, no hepatosplenomegaly, no palpable masses

Skin: faint erythematous maculopapular rash on trunk and in diaper region, no jaundice

**Labs/Radiology:**

- Labs: ↑WBC ↑plts ↑ESR; normal BMP; mild ↑LFTs, ↑ total bilirubin, ↑GGT; normal U/A
- Echocardiogram: Normal coronary artery morphology and LV function

**Patient Summary**

| **Global Assessment or One Liner:** | |
| --- | --- |
|  | |
| **Problem 1: Kawasaki Disease (remember either SOAP or ASOP, including pertinent subjective information and objective information, an assessment and plan)** | |
|  |  |
| **Problem 2: Belly pain** | |
|  |  |

| **Problem 3: Poor appetite** | |
| --- | --- |
|  |  |
