# Supplementary material for: Patient and Family-Centered I-PASS SCORE Program: Resident and Advanced Care Provider Training Materials
Source: MedEdPORTAL. 2022 Aug 9;18:11267. doi: 10.15766/mep_2374-8265.11267 (PMC9360201; doi:10.15766/mep_2374-8265.11267)
Supplement: Supplementary file 1 — PFC I-PASS Core Content Module.m4vHealth Literacy Exercise.docxCore Content Module Evaluation.docxResident and Advanced Care Provider Workshop.pptxResident and Advanced Care Provider Facilitator Guide.docxPFC I-PASS Structure Role-Play.docxPFC I-PASS Rounds Role-Play.docxWritten Handoff for Rounds Role-Play.docxObserver Tool for Rounds Role-Play.docxRounds Report Simulation Activity.docxWorkshop Evaluation.docxBad Example of PFCR.mp4Good Example of PFCR.mp4Tips for Conducting Virtual Teaching Sessions.docx [file mep_2374-8265.11267-s001.zip › C. Core Content Module Evaluation.docx]

**Core-Content Module Participant Evaluation**

**Provider Type (circle):** PGY-1 PGY-2 PGY-3 Fellow MS-3	MS-4 ACP Nurse (Years in practice _ )	Other _____________

**Please rate how this module met its major learning objectives by responding to the following statements:**

| **After completing the module,**  **I am able to:** | **Strongly disagree** | **Disagree** | **Neutral** | **Agree** | **Strongly agree** |
| --- | --- | --- | --- | --- | --- |
| **Describe** the **components** of the PFC  I-PASS training |  |  |  |  |  |
| Describe the impact of **health literacy on health** |  |  |  |  |  |
| **Appreciate** the impact of **Family Centered Rounds** on families and patient care |  |  |  |  |  |
| **List** the key pearls of **health literacy** |  |  |  |  |  |
| **Utilize** **I-PASS structured communication** as an organizing framework for Family Centered Rounds |  |  |  |  |  |
| **Please rate the overall content, structure, pace, and length of the module by responding to the following statements:** | | | | | |
| **This module:** | **Strongly disagree** | **Disagree** | **Neutral** | **Agree** | **Strongly agree** |
| Provided me with knowledge and skills relevant to my patient care activities |  |  |  |  |  |
| Had an appropriate pace |  |  |  |  |  |
| Seemed to be the correct length to address the content |  |  |  |  |  |

**List the most effective elements of the core-content module.**

**Provide specific comments to improve this module.**

**Would you have preferred this content to be delivered in person (circle)? Yes	No	Unsure**
