# Supplementary material for: Patient and Family-Centered I-PASS SCORE Program: Resident and Advanced Care Provider Training Materials
Source: MedEdPORTAL. 2022 Aug 9;18:11267. doi: 10.15766/mep_2374-8265.11267 (PMC9360201; doi:10.15766/mep_2374-8265.11267)
Supplement: Supplementary file 1 — PFC I-PASS Core Content Module.m4vHealth Literacy Exercise.docxCore Content Module Evaluation.docxResident and Advanced Care Provider Workshop.pptxResident and Advanced Care Provider Facilitator Guide.docxPFC I-PASS Structure Role-Play.docxPFC I-PASS Rounds Role-Play.docxWritten Handoff for Rounds Role-Play.docxObserver Tool for Rounds Role-Play.docxRounds Report Simulation Activity.docxWorkshop Evaluation.docxBad Example of PFCR.mp4Good Example of PFCR.mp4Tips for Conducting Virtual Teaching Sessions.docx [file mep_2374-8265.11267-s001.zip › E. Resident and Advanced Care Provider Facilitator Guide.docx]

**Resident/Advanced Practice Provider Training**

**Facilitator Guide**

This guide contains instructions for facilitating and suggested answers for the different interactive elements of the Patient and Family Centered I-PASS training as well as guidance for virtual delivery of the curriculum. It includes:

1. Health Literacy Exercise (completed as part of the core-content module)

Learners should come with the printed documents

2. I-PASS Structure Presentation Simulation (review of the core-content module)

One packet for each learner, divided into participant 1, 2, 3, 4. There are 2 cases, each with an intern presenter, a senior supervisor, and two observers with an observation tool and medical information in the packet

3. I-PASS Rounds Role Play Simulation

One script for each role (7 total)

7 copies of the written handoff (one for each participant)

Observer Tools for those not participating, with observers each given one of 4 areas to focus on

4. Rounds Report Exercise

One packet for each learner

5. Tips for Conducting Virtual Teaching Sessions

Guidelines for the workshop overall as well as each interactive exercise

**Exercise 1 - Health Literacy Exercise – Facilitator Guide**

***Learners should be bringing the completed exercise to the workshop***

**Instructions:**

**Part 1:** You are taking care of Tom Sawyer (TS), just admitted with suspected Kawasaki disease (KD). His mother was with him on admission and now his father has arrived – the father is asking you to give an update and explain what happened – Mrs. Sawyer is there too but is asking you to explain to her husband. You have the history of present illness (HPI) – please convert it into a more health literate update for the father.

Remember:

- Use plain “living-room” language, avoiding medical jargon
- Organize information into 2-3 key concepts (“chunk”)
- Incorporate teach-back to demonstrate shared understanding (“check”)

**Formal History of Present Illness (HPI):**

TS is a 2 year old boy who presents to the primary care clinic with a history of rash, neck swelling, and fever for the past four days. History reveals that he has had double quotidian fever pattern over the past four days. He has been irritable and had a rash that is particularly prominent in the groin and on the trunk. His mother noted a swelling on the right side of his neck 3 days ago. She also noted erythema of both eyes for the past 4 days. In addition, she reports that he has had erythematous, dry, fissured lips, an erythematous tongue with a white exudate, and puffiness on the dorsal aspect of his hands and feet. On examination, the patient had all of the cardinal features of Kawasaki disease (KD), including a 2x3 centimeter enlarged lymph node in the right anterior cervical chain, an erythematous maculo-papular rash involving the trunk and upper extremities, swelling on the dorsal aspects of hands and feet, fissured erythematous lips, and a white strawberry tongue. Laboratory values include a leukocytosis, thrombocytosis, and an elevated erythrocyte sedimentation rate. He is admitted with a presumptive diagnosis of KD and will receive Intravenous Immunoglobulin (IVIG) this evening.

**Update for Father:**

Tom has had spiking fevers, rash, and swelling in his neck for 4 days. His rash is red dots and blotches all over his body and is redder in his diaper area and over his belly. 3 days ago, his mother noticed that his eyes were red. He also has had dry, cracked, red lips and a coated tongue. His feet and hands looked puffy. **Mrs. Sawyer—does that sound about right? Did I leave anything out?**

When we examined Tom when he came into the hospital, we found that he had a fever, a swollen gland on the right side of his neck, a blotchy red rash, swelling on the back of his hands and feet, and dry, cracked lips and a white coating on his tongue. **Mrs. Sawyer—these were the changes you had also noticed in Tom at home, is that right?**

All of these changes are part of an illness called Kawasaki Disease. Kawasaki Disease causes inflammation of blood vessels and can affect the blood vessels of the heart. We treat Kawasaki Disease with Immunoglobulin given through an IV, commonly called IVIG. IVIG has antibodies from the blood which help the body fight inflammation. **Mr. Sawyer, I would like to make sure I did a good job explaining that. Can you tell me how we plan to treat Tom’s Kawasaki Disease?**

**Part 2:** You are now preparing for family centered rounds the next day and developing the patient summary – please use the HPI on the previous page and the overnight updates to draft a health literate patient summary for rounds, focusing on Tom’s 3 problems as listed on the worksheet.

**Night Float Resident Report**

Tom completed his IVIG last night and continues to receive high-dose aspirin four times a day. He had a fever overnight, which came down with one dose of acetaminophen. The team was called twice for abdominal pain, but found Tom’s abdomen to be soft, non-tender, and non-distended. They considered obtaining an abdominal X-ray (KUB) but opted to continue to monitor and reevaluate in the morning.

**Nurse & Mom Overnight Report**

Both Tom’s mother and nurse report that Tom seems less cranky this morning, although he was complaining of a bellyache throughout the night. Tom’s mother reports that there were lots of tears when he cried. He has not had any diarrhea or vomiting. He has had poor oral intake, drinking just sips of Pedialyte from his sippy cup and refusing dinner and breakfast. Tom’s mother thinks his rash and cracked lips are improving.

**Pre-Rounding Data**

**Vital Signs:**

Tmax 39.5^o^C; Tc 37^o^C; HR 80-130bpm; RR 22-30 breaths/min; BP 74/45-110/60 mmHg; Pox 98-100% in room air

Input= 1050cc (maintenance IV fluids); Output = 500cc = 1.5 cc/kg/hr

**Physical Exam:**

GEN: sleeping comfortably in bed

HEENT: normocephalic, atraumatic; conjunctivae without hyperemia or discharge; no scleral icterus; nares patent; lips red without cracking; mucous membranes moist; drool noted on chin

Cardiac: normal S1S2, regular rate and rhythm, no murmur, rub, gallop

Lungs: clear to auscultation bilaterally, no wheeze, rales, rhonchi

Abd: non-distended, + bowel sounds, soft, non-tender, no hepatosplenomegaly, no masses

Skin: faint erythematous maculopapular rash on trunk and in diaper region, no jaundice

**Labs/Radiology:**

- Labs: ↑WBC ↑plts ↑ESR; normal BMP; mild ↑LFTs, ↑ total bilirubin, ↑GGT; normal U/A
- Echocardiogram: Normal coronary artery morphology and LV function.

**Patient Summary**

| **Global Assessment or One Liner:** | |
| --- | --- |
| Tom is a 2 year male admitted with now 5 days of fever, red rash, a swollen gland, swelling of his hands and feet, and cracked lips and coated tongue. Based on these findings, he was diagnosed with Kawasaki Disease. | |
| **Problem 1: Kawasaki Disease (remember either SOAP or ASOP, including pertinent subjective information and objective information, an assessment and plan)** | |
|  | Tom seems to be doing somewhat better today after treatment with IVIG.  Mrs. Sawyer, you and Tom’s nurse said that he is less cranky this morning. On his exam, the rash on his trunk and in his diaper area is fading, and his lips and tongue look better. He still has puffy hands and feet and a swollen gland in his neck, but that is to be expected. He also still had a fever overnight, but we are reassured that this morning he had a normal temperature. On his blood work, his white blood cells, platelets, and ESR were still high—all of these measure inflammation in the body—but we also expect this during the course of Kawasaki Disease, so we are not concerned.  As we discussed yesterday, one important thing we look for in Kawasaki Disease is involvement of the heart. The good news is that we hear no murmur on his heart exam, and the Echocardiogram that Cardiology did yesterday was normal. This means we don’t see any inflammation of the blood vessels in his heart.  So, Tom completed his IVIG treatment yesterday. We are going to continue him on high dose aspirin for now. We will switch him to a lower dose of aspirin when he has been without a fever for 2-3 days. |
| **Problem 2: Belly pain** | |
|  | Tom has had some belly pain. We think Tom’s belly pain may be related to his Kawasaki Disease, as this can sometimes be one of the associated symptoms.  Mrs. Sawyer, you told us that Tom’s stomach started hurting him overnight, but he did not have any diarrhea or vomiting. Last night and this morning, we found his belly exam to be reassuring. His belly was soft, he had normal bowel sounds, and there was no enlargement of his liver or gallbladder. We did note this morning that there was a small increase in his liver enzymes and bilirubin level. This can be another sign of inflammation in Kawasaki’s disease.  Because of this, we want to recheck his liver tests tomorrow morning and check on his belly exam throughout the day. If anything changes, we will get an X-ray of his belly or an ultrasound of his liver and gallbladder to take a closer look. |
| **Problem 3: Poor Appetite** | |
|  | Tom is still not eating or drinking very well, but we have been keeping him well-hydrated with IV fluids.  Even though Tom is only drinking sips of Pedialyte, we are not concerned that he is dehydrated because his lips and tongue are moist, and he is drooling and making tears when he cries. His heart rate and blood pressure are good—these can be abnormal when kids are not getting enough fluids, and he is still peeing very well by all of our measurements. His electrolytes—these are measure of the salts in his blood—were also normal.  You can encourage Tom to eat and drink—we’ll start with clear fluids like soup and jello. If he’s not interested, though, you don’t have push it, we’ll keep giving him his IV fluids. We’ll also continue to keep a close eye on how much he’s peeing. |

**Exercise 2 -** **I-PASS Structure Presentation Simulation Facilitator Guide**

- **Goal: This exercise will allow learners to apply what they learned from the core-content module about health literacy and presenting following the I-PASS mnemonic.**
- **Have the learners divide into groups of 4** - one will be the primary presenter (for example an intern) and the other will be the supervisor (for example the senior resident); the other two will be observers and then the roles will switch for the second case. The setting is the primary presenter is new to the I-PASS mnemonic for presenting and is hoping the senior can help them practice before rounds. It should be practiced as if they are presenting on family centered rounds, using plain language and following the I-PASS mnemonic, including the senior giving the synthesis since there is not a family member at their practice session. The observers will use a tool in the packet to evaluate adherence to the I-PASS presentation elements.
- **Each case has a packet of medical information** – H+P, handoff sheet, and overnight events that the primary presenter will use to develop an I-PASS format presentation for morning rounds - the senior will give the synthesis as well as feedback on the case.
- **Faculty members** (these are people who have been trained as experts – they may be attendings as well as the nurse leads) should observe the groups and facilitate a debrief of the exercise. Faculty should allow participants to self-reflect on how the experience felt, what went well and what were opportunities for improvement, solicit input from the observers using the tool and then faculty will add observations and feedback as needed (the learners should be the primary speakers).
- If there are **family members** participating in the training, we would encourage you to use them as faculty observers to give the learners feedback on the health literacy of the presentation and their thoughts from a family perspective. This is often an incredibly valuable part of the training for learners to get to interact with families in this way.
- If there are **advanced practice providers** participating in the training, they should also be paired to practice the primary presenter role and the senior role.
- The exercise should take about 5 minutes for the presentation and 10 minutes for discussion for each case, with 2 cases total for a total of 30 minutes.

**Exercise 3 - I-PASS Rounds Role Play Simulation Facilitator Guide**

- **Goal: To familiarize team members with the varying roles in PFC-IPASS.**
- **Choose 7 volunteers** (nursing and family advocates assisting with the training can be included) to participate in rounds – there is a role for the attending, nurse, parent and patient, two interns (or medical students, nurse practitioners or other front-line providers) and a senior resident
- **Give each participant the sheet with their role as well as a copy of the handoff document**
- **Each packet has a list of behaviors** the person should display in the role – some things that they do well and some challenges - as well as the medical information for the case (see the following page for a summary). These replicate the frames that people may hold in real life that can be barriers to patient and family centered rounds or promote success.
- **Distribute observer forms to those not participating in the simulation and divide the observers into 4 groups. Each group will be assigned one category to focus on from the observer sheet (I-PASS presentation structure, health literacy, family, and nursing).**
- **The key to success in facilitating simulation exercises is preparing the participants** and observers to create a safe environment to discuss what was seen.
- **The selected group of 7 volunteers will perform the role plays as scripted** – encourage them to embrace the roles but not to overact. You should huddle with the volunteers to review the information and allow them time to prepare.
- **Prepare the audience** so they know that the participants have been assigned roles and certain behaviors – they should watch carefully thinking about what went well, what challenges to the process were faced and how they might have handled the situation.
- After the demonstration, **conduct a debrief** with the observers and participants:
  - **Debriefing is the most important part of a simulation experience to allow learners to reflect on their actions, evaluate what their frame of mind was, and close performance gaps. Follow the guidelines below, encouraging learner discussion with open ended questions, sharing observations and exploring actions, and creating a safe environment to share.**
    - Start by asking participants how it felt.
    - Open to the rest of the audience to identify what went well and what were challenges with following the I-PASS rounding process (you may want to use a whiteboard to record responses).
    - Ask “*what would they do to overcome those challenges in real life?”.*
    - Ask the observers from different groups to share observations from the form.
    - Some points for discussion if the participants do not mention in the course of the debriefing:
      - Discussing the omission of the nurse speaking second.
      - Redirecting the intern using excess medical jargon.
      - Facilitating the positioning of the rounds participants.
      - Assigning roles, including filling out the rounds report.
      - Asking for the synthesis or teach back from the family in a health literate way.

**Goal:** To familiarize team members with the varying roles in PFC-IPASS.

**Case scenario:** Max is a 10-year-old male with known history of asthma, who was brought to the Emergency Department, accompanied by his mother, Mrs. Carter, with a 4-day history of worsening cough, and fever Tmax 102^0^F. Despite at home treatments with albuterol, Max developed tachypnea and labored breathing. He also has had decreased oral intake and only 3 urinary voids in the last 24 hours. On exam, Max has a temperature of 101.7^0^F, HR of 110 bpm, RR of 32 breaths/min, and pulse oximetry of 90% in room air. He is noted to have crackles and decreased aeration over the left lung base. He is diagnosed with community acquired pneumonia and started on IV ampicillin and supplemental O2 via nasal cannula before being admitted to the medical-surgery unit for further care.

**You are part of the rounds team in the role of the intern serving as the primary presenter**

Please review the case information and your key behaviors as listed below (don’t overact but embrace your role)

- You remember your introductions and to ask the family for their impressions of illness severity
- You start your presentation before allowing the nurse to speak second (but are redirectable by the senior if they intervene)
- You feel like you need to present to the attending since evaluations will be completed soon
- You have a hard time with not using medical jargon
- You ask the family to synthesize in a non-health literate way

**You are part of the rounds team in the role of the senior resident**

Please review the case information and your key behaviors as listed below (don’t overact but embrace your role)

- You notice your intern forgot to let the nurse speak second so you try to redirect
- You think the intern’s presentation has gone really well so you don’t need to address anything
- You are trying to make sure the other intern remembers to do the rounds report – you redirect if they are not doing it

**You are part of the rounds team in the role of the intern who is not presenting**

Please review the case information and your key behaviors as listed below (don’t overact but embrace your role)

- You are not sure what your role is other than listening
- You complete the rounds report when your senior resident reminds you and give it to the family at the end

**You are part of the rounds team in the role of the attending**

Please review the case information and your key behaviors as listed below (don’t overact but embrace your role)

- You realize that the intern is not looking at the family and you need to redirect
- You are a little concerned with the amount of medical jargon in the presentation and need to try to redirect the intern
- You are wondering if the family is comfortable synthesizing

**You are part of the rounds team in the role of the nurse**

Please review the case information and your key behaviors as listed below (don’t overact but embrace your role)

- You know you are supposed to share the overnight events after the introductions but don’t know what to do when the intern keeps talking
- You do share if the senior resident redirects the intern or if the intern gets up to the first problem without stopping

**You are part of the rounds team in the role of the parent**

Please review the case information and your key behaviors as listed below (don’t overact but embrace your role)

- You are relieved that Max is doing better overall although his breathing isn’t perfect
- You don’t quite know how to give the synthesis – it feels like a quiz

**You are part of the rounds team in the role of the patient**

Please review the case information and your key behaviors as listed below (don’t overact but embrace your role)

- You are a typical pre-teen – not very verbal but you do answer questions when asked
- You are feeling better, kind of thirsty but not very hungry. You are still having some trouble breathing but are better overall

**You are acting in the role of audience observer**

Please utilize the observer tool on the next page as you watch the role-play

| **GROUP 1 – I-PASS STRUCTURE**  Element | Behavior | Not at all | To a small extent | To a moderate extent | To a great extent |
| --- | --- | --- | --- | --- | --- |
| **Illness Severity**  Identification of patient as better, worse, or about the same  Sharing of concerns  Sharing of overnight events | Family invited to share illness severity and concerns |  |  |  |  |
| **Patient Summary**  Patient one liner: brief pertinent history (where appropriate), and updates from past 24 hours  Assessment and overall plan for hospitalization | One liner statement made |  |  |  |  |
|  | Problem based discussion with structure such as SOAP or ASOP |  |  |  |  |
|  | Overnight events, pertinent positives and negatives shared |  |  |  |  |
|  | Clear assessment, showing clinical reasoning |  |  |  |  |
| **Action List**  Specific action items for next 12-24 hours | Presenter states action items limited to next 12-24 hrs with clear ownership and timeline |  |  |  |  |
| **Situation Awareness and Contingency Planning**  Things to look out for  Anticipating what might happen | Presenter states contingency using if/then language where appropriate |  |  |  |  |
| **Synthesis by Receiver**  Confirming shared understanding amongst team members | Presenter asks family to share their synthesis |  |  |  |  |
|  | Family able to perform synthesis |  |  |  |  |
| **GROUP 2 - Health Literacy – Key Behaviors** | | | | | |
| Utilized plain language terms (avoided medical jargon) | |  |  |  |  |
| Diagnosis (or differential if relevant) explained to family | |  |  |  |  |
| Communication on rounds appropriate for patient/family | |  |  |  |  |
| **GROUP 3 – Family Observations** | | | | | |

| 1 | 2 | 3 | 4 | 5 |
| --- | --- | --- | --- | --- |
| Family not directly addressed at all during presentation. Eye contact with family poor. | Family addressed at end or only minimally during presentation. Very little eye contact made. | Family addressed at times during presentation. Eye contact sometimes made. | Family included in majority of presentation; questions and concerns addressed. Good eye contact maintained for majority of presentation. | Family completely included in presentation; questions and concerns addressed. Eye contact maintained throughout entirety of presentation. **Plan made with family’s input.** |
| 1 | 2 | 3 | 4 | 5 |
| Family present but silent throughout rounds | Family participating minimally during rounds (e.g., answers questions with one- word answers only) | Family somewhat engaged in rounds –asks or responds to questions or speaks up to fill in missing details only when prompted | Family engaged in rounds. May need some prompting, but not always, to answer questions, ask questions, and speak up to fill in missing details | Family completely engaged in rounds. Answered questions, asked questions, and spoke up to fill in missing details and correct misinformation without prompting |

| **GROUP 4 – Nurse Observations** |  |
| --- | --- |

| 1 | 2 | 3 | 4 | 5 |
| --- | --- | --- | --- | --- |
| Nurse not directly addressed at all during presentation. | Nurse addressed only in passing or at end of rounds. | Nurse invited to share second, but otherwise not included in rounds. | Nurse partly included throughout presentation. | Nurse fully included throughout presentation and plan made with nurse’s input. |
| 1 | 2 | 3 | 4 | 5 |
| Nurse present but not paying attention to rounds. May be engaged in other activities. | Nurse present and generally paying attention to rounds but silent. | Nurse present and generally paying attention to rounds. Answers questions, asks questions, and speaks up to fill in missing details but **only when prompted.** | Nurse present and paying attention to rounds. **May need some prompting –but not always**—to answer questions, ask questions, and speak up to fill in missing details. | Nurse present and paying attention to rounds. Answers questions, asks questions, and speaks up to fill in missing details and correct misinformation **without prompting**. |

**Exercise 4 - Rounds Report Tool Exercise**

**Learners are given the following patient information and asked to convert it to a rounds report.**

**A sample completed document follows:**

**Patient Information:** Melissa is a 3yo girl admitted overnight for wheezing. She was made NPO because of her significantly increased work of breathing, and started on continuous albuterol at 5mg/h, and weaned to 2.5mg/h by morning. The overnight team signs out that they think Melissa can be transitioned to albuterol via metered dose inhaler (MDI) every 2 hours. They also think the IV can be locked and she can start some fluids PO.

**Fill out the rounds report tool on the following page using this information.**

**Tips for Conducting Virtual Teaching Sessions**

**Planning considerations:**

If sessions are being done virtually, there are some important considerations for successful planning.

I. Consider the number of participants. While it may feel like you can project to an unlimited number of participants, it becomes hard to maintain engagement for interactive elements if the group is too large. The session should be kept as close to **20 learners** as possible.
II. Virtual sessions may be synchronous and done in real time, or asynchronous with each learner viewing separately. If conducting the in person workshop virtually it should be as a **synchronous session**, rather than just a recorded stream, to allow the learners to ask questions as they process the changes to the rounds structure presented as a workshop focus. Some of the elements may be able to be done asynchronously (see Table 1).
III. For the virtual workshop session, having **two facilitators** present is highly recommended. This allows one person to monitor the chat function as well as setting up the breakout rooms for the interactive elements. Not having a second facilitator will add about 30 minutes to the session overall with preparation for the breakout groups.
IV. Some of the interactive breakout room elements will require additional faculty for facilitation (see Table 1) to achieve a 1:4 ratio.
V. Allow extra time to test the technology – whether videos will play while you are sharing your screen for example. Different platforms (e.g. Zoom and WebEx) have different options for breakout sessions which may require some practice to arrange as the session leader with your IT staff. Other elements to practice and familiarize yourself with include screen sharing, limits on participants, and the chat function. Plan to do a practice session before doing the live teaching session.
VI. If your learners are not on site, they may need some orientation to the platform (e.g. software may need to be downloaded the first time, how to operate the mute function and camera functions, and screen sharing) as well as to ground rules of the session. Important ground rules for the session may include maintaining the camera on, raising a hand with questions, and using the chat function.

**Table 1. Training Material Considerations for Delivery**

| **Workshop Component** | **Delivery** | **Facilitators** | **Time Estimate** | **Other Notes** |
| --- | --- | --- | --- | --- |
| **Health Literacy Exercise** | Synchronous or Asynchronous | 1 for large group share or discussion board monitoring if asynchronous | 5-10 minutes if completed in advance of session | Requires breakout rooms, can be randomly assigned. |
| **I-PASS Presentation Simulation** | Synchronous | 1 per 2-4 learners | 30 minutes | Requires breakout rooms, helpful to be pre-assigned. Roles can be emailed in advance; may need technical support to arrange rooms.  Could be done in small in person groups as part of just- in- time training. |
| **Rounds Simulation:** | Synchronous | 2 facilitators for debriefing | 30 minutes | Plan to email participants in advance. |
| **Rounds Report Exercise** | Synchronous | 1 facilitator for review of content | 5-10 minutes | Could be done in small in-person groups as just-in- time training. |

**Workshop:**

**I. Health Literacy Exercise:**

- Overview:
  - This is a review of the core-content module. Learners are expected to attend the in-person session with the completed document printed.
- Virtual training recommendations:
  - If using a platform that allows for break-out groups (e.g. Zoom):
    - On the day of the workshop, learners could “pair” and review each other’s documents, followed by faculty facilitated sharing with the group at large.
    - A co-facilitator can help assign breakout rooms while the other is presenting slides – for this exercise the pairs can be random
    - If increased time available learners could work together to develop the example at the session in break out groups
  - If using screen sharing:
    - A few selected learners could share their document with the large group to obtain feedback.
  - If using an asynchronous format through a learning management system (e.g. Blackboard):
    - Small groups can be created to review each other’s documents and give feedback in a discussion board format, moderated by faculty and completed prior to the date selected for the session.

**II. I-PASS Presentation Simulation:**

- Overview:
  - This allows practice with the I-PASS format of presentation, done in pairs during the in-person workshop with a faculty monitoring.
- Virtual training recommendations:
  - This session needs to be done in a synchronous fashion either as part of the workshop or as a small group exercise prior to starting on the inpatient unit or ideally both.
  - There are packets for each participant (1-4) containing instructions for participant 1 or 2 for one role play and the observer role for the other role play – these should be emailed in advance of the workshop with instructions to print. These can be displayed as links in the chat function if need be on the day of the session.
  - Learners can be broken into small groups to conduct roles plays with faculty facilitators via:
    1. Use of breakout rooms (e.g., Zoom)
    2. Use of multiple weblinks for separate small group sessions
  - A co-facilitator can be assigning groups while the other facilitator is presenting the slides prior to the session to save time. There should be a grid of faculty and learners, printed in advance to facilitate assignments.
  - There needs to be adequate facilitators to ensure a 1:4 ratio – if this is not achieved it will add time to the session as part of the group will have to be on break until a facilitator is available.
  - Facilitators can come just for this portion and can include nurse champions, family champions, senior residents in addition to attending faculty familiar with the training.
  - A grid with resident groups, faculty facilitators and break out rooms should be created and shared with learners as well as printed by faculty.
  - Learners should be made aware that additional facilitators will join for this portion of the session at the start.
  - Encourage all participants to have their web cameras on during the session.
- Additional tip:
  - Use of this module for a brief just-in-time refresher may prove helpful if repeated or done in advance of the participants initial inpatient rotation.

**III. Rounds Simulation:**

- Overview:
  - This exercise helps familiarize everyone with the rounds process.
- Virtual training recommendations:
  - This session needs to be done in a synchronous fashion, ideally with a second facilitator to help monitor the chat function during the debrief.
  - Ask for volunteers to participate in the role play a week before the session. Others who are not acting in the role play will be assigned an observation tool to help during the debriefing with specific elements to look for and share with the group.
  - Email the participants their role in advance; email everyone the observation tool with instructions to print for the session.
  - Allow 5-10 minutes prior to the role play during the workshop for the participants to review and to have time to huddle with the facilitator to ask questions. This can be accomplished by a separate breakout room or by using the chat function privately.
  - During this time the rest of the group should be reviewing the observation tool and their assigned elements of focus – elements can be preassigned or just done quickly before breaking out using the participant list.
  - Remember, as the facilitator, to orient the audience that the participants have been assigned roles and certain behaviors – they should watch carefully thinking about what went well, what challenges to the process were faced and how they might have handled the situation.
  - Debriefing should be conducted as outlined in the facilitator instructions.
  - A second facilitator may help by typing into a slide or word document to share during the discussion.
  - Web cameras should be on for the session.

**IV. Rounds Report Exercise:**

- Overview:
  - This exercise allows practice of the written rounds report
- Virtual training recommendations:
  - Learners should be instructed to open the document electronically and spend 5 minutes working on the document.
  - Faculty facilitated sharing should happen in the large group.
  - This exercise may be useful to repeat if doing just- in- time training with the I-PASS Presentation Simulations so the skill can be reinforced in real time.
