# Supplementary material for: Patient and Family-Centered I-PASS SCORE Program: Resident and Advanced Care Provider Training Materials
Source: MedEdPORTAL. 2022 Aug 9;18:11267. doi: 10.15766/mep_2374-8265.11267 (PMC9360201; doi:10.15766/mep_2374-8265.11267)
Supplement: Supplementary file 1 — PFC I-PASS Core Content Module.m4vHealth Literacy Exercise.docxCore Content Module Evaluation.docxResident and Advanced Care Provider Workshop.pptxResident and Advanced Care Provider Facilitator Guide.docxPFC I-PASS Structure Role-Play.docxPFC I-PASS Rounds Role-Play.docxWritten Handoff for Rounds Role-Play.docxObserver Tool for Rounds Role-Play.docxRounds Report Simulation Activity.docxWorkshop Evaluation.docxBad Example of PFCR.mp4Good Example of PFCR.mp4Tips for Conducting Virtual Teaching Sessions.docx [file mep_2374-8265.11267-s001.zip › I. Observer Tool for Rounds Role-Play.docx]

**Observer Tool: What term best describes the extent to which the behavior or element listed below was observed?**

**GROUP 1 – IPASS STRUCTURE**

| Element | Behavior | Not at all | To a small extent | To a moderate extent | To a great extent |
| --- | --- | --- | --- | --- | --- |
| **Illness Severity**  Identification of patient as better, worse, or about the same  Sharing of concerns  Sharing of overnight events | Family invited to share illness severity and concerns |  |  |  |  |
| **Patient Summary**  Patient one liner: brief pertinent history (where appropriate), and updates from past 24 hours  Assessment and overall plan for hospitalization | One liner statement made |  |  |  |  |
|  | Problem based discussion with structure such as SOAP or ASOP |  |  |  |  |
|  | Overnight events, pertinent positives and negatives shared |  |  |  |  |
|  | Clear assessment, showing clinical reasoning |  |  |  |  |
| **Action List**  Specific action items for next 12-24 hours | Presenter states action items limited to next 12-24 hrs with clear ownership and timeline |  |  |  |  |
| **Situation Awareness and Contingency Planning**  Things to look out for  Anticipating what might happen | Presenter states contingency using if/then language where appropriate |  |  |  |  |
| **Synthesis by Receiver**  Confirming shared understanding amongst team members | Presenter asks family to share their synthesis |  |  |  |  |
|  | Family able to perform synthesis |  |  |  |  |
| **GROUP 2 - Health Literacy – Key Behaviors** | | | | | |
| Utilized plain language terms (avoided medical jargon) | |  |  |  |  |
| Diagnosis (or differential if relevant) explained to family | |  |  |  |  |
| Communication on rounds appropriate for patient/family | |  |  |  |  |
| **GROUP 3 – Family Observations** | | | | | |

| 1 | 2 | 3 | 4 | 5 |
| --- | --- | --- | --- | --- |
| Family not directly addressed at all during presentation. Eye contact with family poor. | Family addressed at end or only minimally during presentation. Very little eye contact made. | Family addressed at times during presentation. Eye contact sometimes made. | Family included in majority of presentation; questions and concerns addressed. Good eye contact maintained for majority of presentation. | Family completely included in presentation; questions and concerns addressed. Eye contact maintained throughout entirety of presentation. **Plan made with family’s input.** |
| 1 | 2 | 3 | 4 | 5 |
| Family present but silent throughout rounds | Family participating minimally during rounds (e.g., answers questions with one- word answers only) | Family somewhat engaged in rounds –asks or responds to questions or speaks up to fill in missing details only when prompted | Family engaged in rounds. May need some prompting, but not always, to answer questions, ask questions, and speak up to fill in missing details | Family completely engaged in rounds. Answered questions, asked questions, and spoke up to fill in missing details and correct misinformation without prompting |

| **GROUP 4 – Nurse Observations** |  |
| --- | --- |

| 1 | 2 | 3 | 4 | 5 |
| --- | --- | --- | --- | --- |
| Nurse not directly addressed at all during presentation. | Nurse addressed only in passing or at end of rounds. | Nurse invited to share second, but otherwise not included in rounds. | Nurse partly included throughout presentation. | Nurse fully included throughout presentation and plan made with nurse’s input. |
| 1 | 2 | 3 | 4 | 5 |
| Nurse present but not paying attention to rounds. May be engaged in other activities. | Nurse present and generally paying attention to rounds but silent. | Nurse present and generally paying attention to rounds. Answers questions, asks questions, and speaks up to fill in missing details but **only when prompted.** | Nurse present and paying attention to rounds. **May need some prompting –but not always**—to answer questions, ask questions, and speak up to fill in missing details. | Nurse present and paying attention to rounds. Answers questions, asks questions, and speaks up to fill in missing details and correct misinformation **without prompting**. |
