# Supplementary material for: Patient and Family-Centered I-PASS SCORE Program: Resident and Advanced Care Provider Training Materials
Source: MedEdPORTAL. 2022 Aug 9;18:11267. doi: 10.15766/mep_2374-8265.11267 (PMC9360201; doi:10.15766/mep_2374-8265.11267)
Supplement: Supplementary file 1 — PFC I-PASS Core Content Module.m4vHealth Literacy Exercise.docxCore Content Module Evaluation.docxResident and Advanced Care Provider Workshop.pptxResident and Advanced Care Provider Facilitator Guide.docxPFC I-PASS Structure Role-Play.docxPFC I-PASS Rounds Role-Play.docxWritten Handoff for Rounds Role-Play.docxObserver Tool for Rounds Role-Play.docxRounds Report Simulation Activity.docxWorkshop Evaluation.docxBad Example of PFCR.mp4Good Example of PFCR.mp4Tips for Conducting Virtual Teaching Sessions.docx [file mep_2374-8265.11267-s001.zip › J. Rounds Report Simulation Activity.docx]

**Rounds Report Tool Exercise**

**Patient Info:**

Melissa is a 3yo girl admitted overnight for wheezing. She was made NPO because of her significantly increased work of breathing, and started on continuous albuterol at 5mg/h, and weaned to 2.5mg/h by morning.

The overnight team signs out that they think Melissa can be transitioned to albuterol via metered dose inhaler (MDI) every 2 hours. They also think the IV can be locked and she can start some fluids PO

**Fill out the rounds report tool on the following page using this information**

S

A

Rounds Report

*“Rounds are when doctors and other team members come in a group in the morning to talk with you/your child. They usually see how you/your child is doing and make a plan for the day with you.”*

I – Illness Severity & Introductions P – Patient Summary

A – Action List

S – Situation Awareness & Contingency Planning

S – Synthesis by Receiver

Date:

Patient name:

You are an important part of the team caring for your child! This form will help you know what will happen today

and how you can help. Use it to take notes. You can also write down what you are worried about and any questions you have for us later in the day or tomorrow on rounds.

I

Why you/your child is in the hospital:

Today you/your child is: (Check appropriate box)	Better	Same	Worse Did you have new concerns on rounds today? (Check appropriate box)	Yes	No

P

| Important Updates  - *What are important, new things for you to know* |  |
| --- | --- |
| What should be done today to help you/your child get better?  - *Plan for the day* |  |
| Things that you can help us look out for; things that you can do today. |  |

Additional notes and information:

-	*Please feel to take notes in this section after rounds.*

©2019, The Children’s Hospital Corporation. For permission to use, please contact [Family.IPASS@childrens.harvard.edu.](mailto:Family.IPASS@childrens.harvard.edu)

Questions or things you would like to talk about tomorrow:

-	*Please fill this section out after rounds in preparation for rounds tomorrow.*
