# Supplementary material for: Patient and Family-Centered I-PASS SCORE Program: Resident and Advanced Care Provider Training Materials
Source: MedEdPORTAL. 2022 Aug 9;18:11267. doi: 10.15766/mep_2374-8265.11267 (PMC9360201; doi:10.15766/mep_2374-8265.11267)
Supplement: Supplementary file 1 — PFC I-PASS Core Content Module.m4vHealth Literacy Exercise.docxCore Content Module Evaluation.docxResident and Advanced Care Provider Workshop.pptxResident and Advanced Care Provider Facilitator Guide.docxPFC I-PASS Structure Role-Play.docxPFC I-PASS Rounds Role-Play.docxWritten Handoff for Rounds Role-Play.docxObserver Tool for Rounds Role-Play.docxRounds Report Simulation Activity.docxWorkshop Evaluation.docxBad Example of PFCR.mp4Good Example of PFCR.mp4Tips for Conducting Virtual Teaching Sessions.docx [file mep_2374-8265.11267-s001.zip › K. Workshop Evaluation.docx]

**Post-Training Participant Evaluation**

**Provider Type (circle):** PGY-1 PGY-2 PGY-3 Fellow MS-3	MS-4 ACP Nurse (Years in practice _ )	Other _____________

**Age (circle):** 20-25	26-30	31-35	36-40	40-45	45-50	**Gender(circle):** He/Him	She/Her	They/Their

**Race (circle):** Asian	Black	Hispanic	White	Other

**Did you complete the core-content module prior to attending the in person training (circle)?** Yes	No

**Please rate how this training met its major learning objectives by responding to the following statements:**

| **After participating in the training,**  **I am able to:** | **Strongly disagree** | **Disagree** | **Neutral** | **Agree** | **Strongly agree** |
| --- | --- | --- | --- | --- | --- |
| **Activate and engage** families and all members of the inter-professional team to create a shared mental model |  |  |  |  |  |
| Apply **health literacy principles** to improve communication |  |  |  |  |  |
| Incorporate **I-PASS structured communication** as an organizing framework for Family Centered Rounds |  |  |  |  |  |
| Demonstrate effective use of **written communication** to facilitate communication with families and the inter-professional team |  |  |  |  |  |
| List appropriate **educational activities** for Family Centered Rounds  (skip question if student) |  |  |  |  |  |
| Discuss the **roles and responsibilities for various team members** during patient and family centered rounds |  |  |  |  |  |
| **Please rate the overall content, structure, pace and length of the workshop by responding to the following statements:** | | | | | |
| **This workshop:** | **Strongly disagree** | **Disagree** | **Neutral** | **Agree** | **Strongly agree** |
| Provided me with knowledge and skills relevant to my patient care activities |  |  |  |  |  |
| Was designed with an appropriate balance of didactic and interactive elements |  |  |  |  |  |
| Had an appropriate pace |  |  |  |  |  |
| Seemed to be the correct length to address the content |  |  |  |  |  |

**List the most effective elements of the overall training.**

**List the most effective elements of the workshop.**

**Provide specific comments to improve this training.**

**Please provide comments about the simulation activities.**
