# Supplementary material for: Patient and Family-Centered I-PASS SCORE Program: Resident and Advanced Care Provider Training Materials
Source: MedEdPORTAL. 2022 Aug 9;18:11267. doi: 10.15766/mep_2374-8265.11267 (PMC9360201; doi:10.15766/mep_2374-8265.11267)
Supplement: Supplementary file 1 — PFC I-PASS Core Content Module.m4vHealth Literacy Exercise.docxCore Content Module Evaluation.docxResident and Advanced Care Provider Workshop.pptxResident and Advanced Care Provider Facilitator Guide.docxPFC I-PASS Structure Role-Play.docxPFC I-PASS Rounds Role-Play.docxWritten Handoff for Rounds Role-Play.docxObserver Tool for Rounds Role-Play.docxRounds Report Simulation Activity.docxWorkshop Evaluation.docxBad Example of PFCR.mp4Good Example of PFCR.mp4Tips for Conducting Virtual Teaching Sessions.docx [file mep_2374-8265.11267-s001.zip › N. Tips for Conducting Virtual Teaching Sessions.docx]

**Tips for Conducting Virtual Teaching Sessions**

**Planning considerations:**

If sessions are being done virtually, there are some important considerations for successful planning.

I. Consider the number of participants. While it may feel like you can project to an unlimited number of participants, it becomes hard to maintain engagement for interactive elements if the group is too large. The session should be kept as close to **20 learners** as possible.
II. Virtual sessions may be synchronous and done in real time, or asynchronous with each learner viewing separately. If conducting the in person workshop virtually it should be as a **synchronous session**, rather than just a recorded stream, to allow the learners to ask questions as they process the changes to the rounds structure presented as a workshop focus. Some of the elements may be able to be done asynchronously (see Table 1).
III. For the virtual workshop session, having **two facilitators** present is highly recommended. This allows one person to monitor the chat function as well as setting up the breakout rooms for the interactive elements. Not having a second facilitator will add about 30 minutes to the session overall with preparation for the breakout groups.
IV. Some of the interactive breakout room elements will require additional faculty for facilitation (see Table 1) to achieve a 1:4 ratio.
V. Allow extra time to test the technology – whether videos will play while you are sharing your screen for example. Different platforms (e.g. Zoom and WebEx) have different options for breakout sessions which may require some practice to arrange as the session leader with your IT staff. Other elements to practice and familiarize yourself with include screen sharing, limits on participants, and the chat function. Plan to do a practice session before doing the live teaching session.
VI. If your learners are not on site, they may need some orientation to the platform (e.g. software may need to be downloaded the first time, how to operate the mute function and camera functions, and screen sharing) as well as to ground rules of the session. Important ground rules for the session may include maintaining the camera on, raising a hand with questions, and using the chat function.

Table 1:

| **Workshop Component** | **Delivery** | **Facilitators** | **Time Estimate** | **Other Notes** |
| --- | --- | --- | --- | --- |
| **Health Literacy Exercise** | Synchronous or Asynchronous | 1 for large group share or discussion board monitoring if asynchronous | 5-10 minutes if completed in advance of session | Requires breakout sessions, can be randomly assigned |
| **I-PASS Presentation Simulation** | Synchronous | 1 per 2-4 residents | 30 minutes | Requires breakout sessions, helpful to be preassigned, roles to be emailed in advance, may need technical support to arrange rooms  Could be done in small in person groups as just in time training |
| **Rounds Simulation:** | Synchronous | 2 facilitators for debriefing | 30 minutes | Plan to email participants in advance |
| **Rounds Report Exercise** | Synchronous |  |  | Could be done in small in person groups as just in time training |

**Workshop:**

**I. Health Literacy Exercise:**

- Overview:
  - This is a review of the core content pre-work. Students are expected to attend the in-person session with the printed document.
- Virtual training recommendations:
  - If using a platform that allows for break-out groups (e.g. Zoom):
    - On the day of the workshop, students could “pair” and review each other’s documents, followed by faculty facilitated sharing with the group at large.
    - A co-facilitator can help assign breakout rooms while the other is presenting slides – for this exercise the pairs can be random
    - If increased time available students could work together to develop the example at the session in break out groups
  - If using screen sharing:
    - A few selected students could share their document with the large group to obtain feedback.
  - If using an asynchronous format through a learning management system (e.g. Blackboard):
    - Small groups can be created to review each other’s documents and give feedback in a discussion board format, moderated by faculty and completed prior to the date selected for the session.

**II. I-PASS Presentation Simulation:**

- Overview:
  - This allows practice with the I-PASS format of presentation, done in pairs during the in-person workshop with a faculty monitoring.
- Virtual training recommendations:
  - This session needs to be done in a synchronous fashion either as part of the workshop or as a small group exercise prior to starting on the inpatient unit or ideally both
  - There are packets for each participant (1-4) containing instructions for participant 1 or 2 for one role play and the observer role for the other role play – these should be emailed in advance of the workshop with instructions to print. These can be displayed as links in the chat function if need be on the day of the session.
  - Students can be broken into small groups to conduct roles plays with faculty facilitators via:
    1. Use of breakout sessions (e.g. Zoom)
    2. Use of multiple weblinks for separate small group sessions
  - A co-facilitator can be assigning groups while the other facilitator is presenting the slides prior to the session to save time. There should be a grid of faculty and learners, printed in advance to facilitate assignments
  - There need to be adequate facilitators to ensure a 1:4 ratio – if this is not achieved it will add time to the session as part of the group will have to be on break until a facilitator is available
  - Facilitators can come just for this portion and can include nurse champions, family champions, senior residents in addition to attending faculty
  - A grid with resident groups, faculty facilitators and break out group should be created and shared with learners as well as printed by faculty.
  - Learners should be made aware that additional facilitators will join for this portion of the session at the start.
  - Encourage all participants to have their web cameras on during the session.
- Additional tips:
  - Use of this module for a brief just-in-time refresher may prove helpful if repeated or done in advance of the participants initial inpatient rotation.

**III. Rounds Simulation:**

- Overview:
  - This exercise helps familiarize everyone with the rounds process.
- Virtual training recommendations:
  - This session needs to be done in a synchronous fashion, ideally with a second facilitator to help monitor the chat function during the debrief.
  - Ask for volunteers to participate in the role play a week before the session. Others who are not acting in the role play will be assigned an evaluation tool to help during the debriefing with specific elements to look for and share with the group.
  - Email the participants their role in advance; email everyone the observation tool with instructions to print for the session.
  - Allow 5-10 minutes prior to the role play during the workshop for the participants to review and to have time to huddle with the facilitator to ask questions. This can be accomplished by a separate breakout session or by using the chat function privately.
  - During this time the rest of the group should be reviewing the observation tool and their assigned elements of focus – elements can be preassigned or just done quickly before breaking out using the participant list.
  - Remember, as the facilitator, to orient the audience that the participants have been assigned roles and certain behaviors – they should watch carefully thinking about what went well, what challenges to the process were faced and how they might have handled the situation.
  - Debriefing should be conducted as outlined in the facilitator instructions.
  - Second facilitator may help by typing into a slide or word document to share during the discussion.
  - Web cameras should be on for the session.

**IV. Rounds Report Exercise:**

- Overview:
  - This exercise allows practice of the written rounds report
- Virtual training recommendations:
  - Learners should be instructed to open the document electronically and spend 5 minutes working on the document.
  - Faculty facilitated sharing should happen in the large group.
  - This exercise may be useful to repeat if doing just in time training with the I-PASS Presentation Simulations so the skill can be reinforced in real time.
