# Supplementary material for: Patient and Family-Centered I-PASS SCORE Program: Resident and Advanced Care Provider Training Materials
Source: MedEdPORTAL. 2022 Aug 9;18:11267. doi: 10.15766/mep_2374-8265.11267 (PMC9360201; doi:10.15766/mep_2374-8265.11267)
Supplement: Supplementary file 1 — PFC I-PASS Core Content Module.m4vHealth Literacy Exercise.docxCore Content Module Evaluation.docxResident and Advanced Care Provider Workshop.pptxResident and Advanced Care Provider Facilitator Guide.docxPFC I-PASS Structure Role-Play.docxPFC I-PASS Rounds Role-Play.docxWritten Handoff for Rounds Role-Play.docxObserver Tool for Rounds Role-Play.docxRounds Report Simulation Activity.docxWorkshop Evaluation.docxBad Example of PFCR.mp4Good Example of PFCR.mp4Tips for Conducting Virtual Teaching Sessions.docx [file mep_2374-8265.11267-s001.zip › F. PFC I-PASS Structure Role-Play.docx]

**Patient and Family Centered I-PASS Role Plays**

**I-PASS Structure Presentation Role Play**

- **The objective of these role plays is to allow you to practice the I-PASS structure of a rounds presentation as reviewed in the core-content module.**
- **In one role play you are an observer and in one you are a participant**
- **As a participant:**
  - **Please follow the instructions carefully that explain your role and embrace that given role.**
  - **Please review the instruction sheet and the packet of clinical information you receive and present as directed.**
  - **If medical information is missing please feel free to add what is necessary.**
- **As an observer:**
  - **Please use the observation tool provided to look for key elements and behaviors that are part of the I-PASS Presentation**
  - **Be prepared to share your observations in the debrief of the role play**

I

Illness Severity

Introduce Team Members and FCR

Getting better, worse, the same

P

Patient Summary

Problem Oriented

Ongoing Assessment and Plan

A

Action List

To Do List

S

Situational Awareness and Contingency Planning

Knowing what's going on

Planning for what might happen

S

Synthesis by Receiver

Teach back, receiver summarizes what was heard, encourage family to perform

Health Literacy

Teach Back

Plain Language

Slow Down!

Facilitate Conversation

Embrace parent role

Chunk and check

**Observer Tool**

**What term best describes the extent to which the behavior or element listed below was observed?**

| Element | Behavior | Not at all | To a small extent | To a moderate extent | To a great extent |
| --- | --- | --- | --- | --- | --- |
| **Illness Severity**  Identification of patient as better, worse, or about the same  Sharing of concerns  Sharing of overnight events | Family invited to share illness severity and concerns |  |  |  |  |
| **Patient Summary**  Patient one liner: brief pertinent history (where appropriate), and updates from past 24 hours  Assessment and overall plan for hospitalization | One liner statement made |  |  |  |  |
|  | Problem based discussion with structure such as SOAP or ASOP |  |  |  |  |
|  | Overnight events, pertinent positives and negatives shared |  |  |  |  |
|  | Clear assessment, showing clinical reasoning |  |  |  |  |
| **Action List**  Specific action items for next 12-24 hours | Presenter states action items limited to next 12-24 hours with clear ownership and timeline |  |  |  |  |
| **Situation Awareness and Contingency Planning**  Things to look out for  Anticipating what might happen | Presenter states contingency using if/then language where appropriate |  |  |  |  |
| **Synthesis by Receiver**  Confirming shared understanding amongst team members | Presenter asks family to share their synthesis, but Senior demonstrates synthesis |  |  |  |  |
|  | Synthesis is brief restating key points, not a restatement of whole presentation |  |  |  |  |
| **Health Literacy – Key Behaviors** | |  |  |  |  |
| Utilized plain language terms (avoided medical jargon) | |  |  |  |  |
| Diagnosis (or differential if relevant) explained to family | |  |  |  |  |
| Communication on rounds appropriate for patient/family | |  |  |  |  |
| **Summary** | |  |  |  |  |
| What was the most effective thing you observed about the presentation? | |  | | | |
| What is the most important thing that could be done to improve the presentation? | |  | | | |
| Additional Comments | |  | | | |

**Case 1**

**Participant 1 – Primary Presenter**

You are just starting the inpatient service today and have picked up the overnight admissions. You are trying to learn the patients quickly before rounds. Your program has recently initiated family centered rounds. You will be presenting first. You are nervous because the program director is the inpatient attending and you want to make a good impression. You have been to the training but have not yet presented in this way. Your senior has offered to have you practice before rounds.

- You are expected to present to your senior in the full I-PASS format for Patient and Family Centered Rounds.
- Be mindful of health literacy in your presentation
- Remember you will ultimately be in front of the team, including a parent – embrace this when you are presenting

**Participant 2 – Senior Supervisor**

You are just starting the inpatient service today and have picked up the overnight admissions. Your program has recently initiated family centered rounds and your program director is on service. You realize none of the team members have presented in the I-PASS structure before and offer to help them practice before rounds start. You really want them to demonstrate a health literate approach in the presentation. You will role model the synthesis portion as there is no family present.

- Pay attention to your junior’s presentation
- Be mindful of health literacy in their presentation
- Remember you will ultimately be in front of the team, including a parent – embrace this when you are presenting

**Participant 3 - Observer Role**

You are observing an intern and senior practicing their I-PASS presentation before morning rounds. Please use the observation form on the next page and look for key elements of the presentation. Be prepared to share your observations at the end. Medical information about the case is on the following page if needed.

**Participant 4 - Observer Role**

You are observing an intern and senior practicing their I-PASS presentation before morning rounds. Please use the observation form on the next page and look for key elements of the presentation. Be prepared to share your observations at the end. Medical information about the case is on the following page if needed.

**Medical Information for Role Play Case 1**

**Case 1**

**Admission H+P**

**CC:** Fever and refusal to walk

**HPI:** CS is a 3 year old girl who has had low grade fever for the past 3 days and now is refusing to walk. Her parents report mild trauma when she fell while dancing on the hardwood floor one week ago. At the time she complained about pain in her left leg, but she had no obvious bruising or swelling. Her mother reports that she never exhibited an antalgic gait but, upon arising this morning, she was unable to bear weight on the left lower extremity. She has otherwise been well with no recent URI, no cough, no vomiting or diarrhea.

**PMH:** None	**PSH:** None	**MED:** None	**ALL:** No known drug allergies

**Physical Exam:**

Vital Signs: T:39 ^0^C HR: 120bpm R:22 breaths/min BP: 100/70 mmHg Pox: 100% in room air

General: Fussy, uncomfortable, lying in bed

HEENT: normocephalic/atraumatic; pupils equal round and reactive to light, extraocular muscles intact; tympanic membranes clear; throat without erythema, no lymphadenopathy

Cardiac: normal S1S2, regular rate and rhythm, no murmur, rub, gallop

Lungs: clear to auscultation bilaterally, no wheeze, rales, rhonchi

Abd: non-distended, +bowel sounds, soft, non-tender, no hepatosplenomegaly

GU: Tanner I

Ext: left distal femur with tenderness on deep palpation; no swelling or erythema noted; small healing abrasion lateral thigh above knee; full range of motion at hips and knees; no effusion; right leg normal

Neuro: awake, alert, unhappy; CN 2-12 intact, motor 5/5, no sensory deficits, refuses to ambulate, will not bear weight on left lower extremity

**Labs/Imaging**

WBC 25; Differential 50% segs, 15% bands, 32% lymphocytes, Hgb 10, plts 438

Electrolytes normal

CRP 32 ESR 110

Blood culture: pending

Plain films of bilateral femurs normal

**Assessment:** 3yo female admitted for refusal to bear weight on left leg and fevers, concerning for osteomyelitis

**Plan:** 1. Start IV Clindamycin

2. Orthopedic consult

3. Repeat labs in AM

4. NPO for possible sedated MRI in the morning

| **Case 1 AM Handoff Document** | **I** | **P** | **A** | **S** | **S** |
| --- | --- | --- | --- | --- | --- |
|  | **Illness**  **Severity** | **Patient Summary** | **Action List** | **Situation Awareness &**  **Contingency Planning** | **Synthesis by**  **Receiver** |
| M6E **Sawyer, Claire**  2/23/2019  MRN: 45612378  Visit: 45615  Adm Date: 3/11/22  Attending: Brown, Julie  Code Status: Full  Allergies: NKDA  Wt: 12 kg  Access: PIV  Resident: Cameron, Jack  **Medications** Clindamycin |  | 3yo female presents with 3 days of fever, refusal to walk this morning.  Presented with history mild trauma 1 week ago, febrile x 3 days, now refusal bear weight left leg, concern osteomyelitis.  Leg Pain – elevated inflammatory markers, CBC suggestive of osteomyelitis, on IV Clindamycin | - Follow up cultures - Call MRI to arrange time slot for morning for MRI L femur - Follow up ortho consult - NPO at MN for sedation with MRI - Repeat ESR/CRP in AM | - If has increased pain, then would give morphine and call ortho - If febrile, then re-culture | SYNTHESIS BY RECEIVER |

**Case 1 Overnight Events**

Overnight febrile; Tmax 39.5°C

HR 90-120bpm, BP 92-106/55-62 mmHg, RR 18-24 breaths/min, Pox 100% in room air

Intake:1300cc Output:889cc

No increased pain overnight, cultures sent, labs pending for this AM, MRI scheduled for 11AM

**Observer Tool**

**What term best describes the extent to which the behavior or element listed below was observed?**

| Element | Behavior | Not at all | To a small extent | To a moderate extent | To a great extent |
| --- | --- | --- | --- | --- | --- |
| **Illness Severity**  Identification of patient as better, worse, or about the same  Sharing of concerns  Sharing of overnight events | Family invited to share illness severity and concerns |  |  |  |  |
| **Patient Summary**  Patient one liner: brief pertinent history (where appropriate), and updates from past 24 hours  Assessment and overall plan for hospitalization | One liner statement made |  |  |  |  |
|  | Problem based discussion with structure such as SOAP or ASOP |  |  |  |  |
|  | Overnight events, pertinent positives and negatives shared |  |  |  |  |
|  | Clear assessment, showing clinical reasoning |  |  |  |  |
| **Action List**  Specific action items for next 12-24 hours | Presenter states action items limited to next 12-24 hours with clear ownership and timeline |  |  |  |  |
| **Situation Awareness and Contingency Planning**  Things to look out for  Anticipating what might happen | Presenter states contingency using if/then language where appropriate |  |  |  |  |
| **Synthesis by Receiver**  Confirming shared understanding amongst team members | Presenter asks family to share their synthesis, but Senior demonstrates synthesis |  |  |  |  |
|  | Synthesis is brief restating key points, not a restatement of whole presentation |  |  |  |  |
| **Health Literacy – Key Behaviors** | |  |  |  |  |
| Utilized plain language terms (avoided medical jargon) | |  |  |  |  |
| Diagnosis (or differential if relevant) explained to family | |  |  |  |  |
| Communication on rounds appropriate for patient/family | |  |  |  |  |
| **Summary** | |  |  |  |  |
| What was the most effective thing you observed about the presentation? | |  | | | |
| What is the most important thing that could be done to improve the presentation? | |  | | | |
| Additional Comments | |  | | | |

**Case 2**

**Participant 1 - Observer Role**

You are observing an intern and senior practicing their I-PASS presentation before morning rounds. Please use the observation form on the next page and look for key elements of the presentation. Be prepared to share your observations at the end. Medical information about the case is on the following page if needed.

**Participant 2 - Observer Role**

You are observing an intern and senior practicing their I-PASS presentation before morning rounds. Please use the observation form on the next page and look for key elements of the presentation. Be prepared to share your observations at the end. Medical information about the case is on the following page if needed.

**Participant 3 – Senior Supervisor**

You are just starting the inpatient service today and have picked up the overnight admissions. Your program has recently initiated family centered rounds and your program director is on service. You realize none of the rounding team have presented in the I-PASS structure before and offer to help them practice before rounds start. You really want them to demonstrate a health literate approach in the presentation. You will do the synthesis at the end to role model this since there is no family present

- Pay attention to your junior’s presentation
- Be mindful of health literacy in their presentation
- Remember you will ultimately be in front of the team, including a parent – embrace this when you are presenting

**Participant 4 – Primary Presenter**

You are just starting the inpatient service today and have picked up the overnight admissions. You are trying to learn the patients quickly before rounds. Your program has recently initiated family centered rounds. You will be presenting first. You are nervous because the program director is the inpatient attending and you want to make a good impression. You have been to the training but have not yet presented in this way. Your senior has offered to have you practice before rounds.

- You are expected to present to your senior in the full I-PASS format for Patient and Family Centered Rounds.
- Be mindful of health literacy in your presentation
- Remember you will ultimately be in front of the team, including a parent – embrace this when you are presenting

**Medical Information for Role Play Case 2**

**Case 2**

**Admission H+P**

**CC:** Vomiting, diarrhea, and fever

**HPI:** KL is a 2 year old girl who presents to the primary care clinic with a history of vomiting, diarrhea, and fever for the past three days. History reveals that she had been in her usual state of health until the onset of symptoms. Her mother first noted the fever on the first day of symptoms, followed by non-bilious vomiting and non-bloody diarrhea the subsequent days. She also noted that KL has been unable to drink or eat, and her lips have become dry. She is uncertain if she is urinating normally, as her bowel movements are quite watery. She denies ingestion of new or raw foods. She also denies exposure to reptiles. KL attends daycare, and there have been other children with similar symptoms.

**PMH:** None	**PSH:** None	**MED:** None	**ALL:** No known drug allergies

**Physical:**

Vital Signs: T:38.5 ^0^C HR: 130bpm R:20 breaths/min BP: 97/60 mmHg Pox: 100% in room air

General: Mildly ill appearing, uncomfortable, sitting in mother’s lap

HEENT: normocephalic/atraumatic; pupils equal round and reactive to light, conjunctiva clear, tympanic membranes clear; lips dry, + tacky mucous membranes

Cardiac: normal S1S2, + tachycardia, no murmur, rub, gallop

Lungs: clear to auscultation bilaterally, no wheeze, rales, rhonchi

Abd: non-distended, + hyperactive bowel sounds, soft, + diffuse tenderness, negative Obturator sign, negative Psoas sign

Neuro: awake, alert, unhappy CN 2-12 intact, motor 5/5, no sensory defects

**Labs**

Na: 131

Stool studies: pending

**Assessment:** 2 yo female admitted for fever with associate non-bilious vomiting and non-bloody diarrhea, concerning for acute gastroenteritis with dehydration

**Plan:**

1. IV hydration until po intake improves

2. Monitor intake and output

3. Acetaminophen as needed for fever

4. Ondansetron as needed for nausea

| **Case 2 AM Handoff Document** | **I** | **P** | **A** | **S** | **S** |
| --- | --- | --- | --- | --- | --- |
|  | **Illness**  **Severity** | **Patient Summary** | **Action List** | **Situation Awareness &**  **Contingency Planning** | **Synthesis by**  **Receiver** |
| M8E **Love, Kim**  3/01/2020  MRN: 7865123  Visit: 8910  Adm Date: 3/11/22  Attending: Brown, Julie  Code Status: Full  Allergies: NKDA  Wt: 9 kg  Access: PIV  Resident: Cameron, Jack  **Medications**  Acetaminophen  Ondansetron |  | 2yo female presents with 3 days of fever, vomiting, and diarrhea  She was noted to have onset of fever followed by non-bilious vomiting and non-bloody diarrhea. Decline in oral intake. Difficult to assess urinary output due to volume and frequency of diarrhea.   1. Dehydration – has had poor po intake, placed on IVF 2. Gastroenteritis- suspected viral, but stool cultures pending, ondansetron given x1 | - Check I+O at 9pm - Monitor fever curve - Follow-up stool culture | - If decreased urine output, then consider giving a 20cc/kg NS bolus | SYNTHESIS BY RECEIVER |

**Case 2 Overnight Events**

Overnight Tmax 39^o^C; Tc 37^o^C; HR 80-130bpm, RR 22-26 breaths/min, BP 74-110/45-60 mmHg Pox 100% in room air

I= 1050cc (maintenance IV fluids); O = 500cc; UOP = 1.5 cc/kg/hr

Improving oral intake overnight, received acetaminophen for fever, continues to have watery diarrhea
