# Supplementary material for: Patient and Family-Centered I-PASS SCORE Program: Resident and Advanced Care Provider Training Materials
Source: MedEdPORTAL. 2022 Aug 9;18:11267. doi: 10.15766/mep_2374-8265.11267 (PMC9360201; doi:10.15766/mep_2374-8265.11267)
Supplement: Supplementary file 1 — PFC I-PASS Core Content Module.m4vHealth Literacy Exercise.docxCore Content Module Evaluation.docxResident and Advanced Care Provider Workshop.pptxResident and Advanced Care Provider Facilitator Guide.docxPFC I-PASS Structure Role-Play.docxPFC I-PASS Rounds Role-Play.docxWritten Handoff for Rounds Role-Play.docxObserver Tool for Rounds Role-Play.docxRounds Report Simulation Activity.docxWorkshop Evaluation.docxBad Example of PFCR.mp4Good Example of PFCR.mp4Tips for Conducting Virtual Teaching Sessions.docx [file mep_2374-8265.11267-s001.zip › G. PFC I-PASS Rounds Role-Play.docx]

**I-PASS Rounds Role Play Simulation Activity**

**Roles included:**

1. Presenting Intern (page 2)
2. Nonpresenting Intern (page 3-4)
3. Parent (page 5)
4. Patient (page 6)
5. Faculty Member (page 7)
6. Nurse (Page 8)
7. Senior Resident (page 9)

**I-PASS Rounds Role Play Simulation Activity**

**Goal:** To familiarize team members with the varying roles in PFC-IPASS.

**You are part of the rounds team in the role of the intern serving as the primary presenter**

Please review the case information and your key behaviors as listed below (don’t overact but embrace your role)

- You remember your introductions and to ask the family for their impressions of illness severity
- You start your presentation before allowing the nurse to speak second (but are redirectable by the senior if they intervene)
- You feel like you need to present to the attending since evaluations will be completed soon
- You have a hard time with not using medical jargon
- You ask the family to synthesize in a non health literate way

**Case scenario:** Max is a 10-year-old male with known history of asthma, who was brought to the Emergency Department, accompanied by his mother, Mrs. Carter, with a 4-day history of worsening cough, and fever Tmax 102°F. Despite at home treatments with albuterol, Max developed tachypnea and labored breathing. He also has had decreased oral intake and only 3 urinary voids in the last 24 hours. On exam, Max has a temperature of 101.7°F, HR of 110 bpm, RR of 32 breaths/min, and pulse oximetry of 90% in room air. He is noted to have crackles and decreased aeration over the left lung base. He is diagnosed with community acquired pneumonia and started on IV ampicillin and supplemental O2 via nasal cannula before being admitted to the medical-surgery unit for further care.

**I-PASS Rounds Role Play Simulation Activity**

**Goal:** To familiarize team members with the varying roles in PFC-IPASS.

**You are part of the rounds team in the role of the intern who is not presenting**

Please review the case information and your key behaviors as listed below (don’t overact but embrace your role)

- You are not sure what your role is other than listening
- You complete the rounds report when your senior resident reminds you and give it to the family at the end

**Case scenario:** Max is a 10-year-old male with known history of asthma, who was brought to the Emergency Department, accompanied by his mother, Mrs. Carter, with a 4-day history of worsening cough, and fever Tmax 102°F. Despite at home treatments with albuterol, Max developed tachypnea and labored breathing. He also has had decreased oral intake and only 3 urinary voids in the last 24 hours. On exam, Max has a temperature of 101.7°F, HR of 110 bpm, RR of 32 breaths/min, and pulse oximetry of 90% in room air. He is noted to have crackles and decreased aeration over the left lung base. He is diagnosed with community acquired pneumonia and started on IV ampicillin and supplemental O2 via nasal cannula before being admitted to the medical-surgery unit for further care.

**I-PASS Rounds Role Play Simulation Activity**

**Goal:** To familiarize team members with the varying roles in PFC-IPASS.

**You are part of the rounds team in the role of the parent**

Please review the case information and your key behaviors as listed below (don’t overact but embrace your role)

- You are relieved that Max is doing better overall although his breathing isn’t perfect
- You don’t quite know how to give the synthesis – it feels like a quiz

**Case scenario:** Max is a 10-year-old male with known history of asthma, who was brought to the Emergency Department, accompanied by his mother, Mrs. Carter, with a 4-day history of worsening cough, and fever Tmax 102°F. Despite at home treatments with albuterol, Max developed tachypnea and labored breathing. He also has had decreased oral intake and only 3 urinary voids in the last 24 hours. On exam, Max has a temperature of 101.7°F, HR of 110 bpm, RR of 32 breaths/min, and pulse oximetry of 90% in room air. He is noted to have crackles and decreased aeration over the left lung base. He is diagnosed with community acquired pneumonia and started on IV ampicillin and supplemental O2 via nasal cannula before being admitted to the medical-surgery unit for further care.

**I-PASS Rounds Role Play Simulation Activity**

**Goal:** To familiarize team members with the varying roles in PFC-IPASS.

**You are part of the rounds team in the role of the patient**

Please review the case information and your key behaviors as listed below (don’t overact but embrace your role)

- You are a typical pre-teen – not very verbal but you do answer questions when asked
- You are feeling better, kind of thirsty but not very hungry. You are still having some trouble breathing but are better overall

**Case scenario:** Max is a 10-year-old male with known history of asthma, who was brought to the Emergency Department, accompanied by his mother, Mrs. Carter, with a 4-day history of worsening cough, and fever Tmax 102°F. Despite at home treatments with albuterol, Max developed tachypnea and labored breathing. He also has had decreased oral intake and only 3 urinary voids in the last 24 hours. On exam, Max has a temperature of 101.7°F, HR of 110 bpm, RR of 32 breaths/min, and pulse oximetry of 90% in room air. He is noted to have crackles and decreased aeration over the left lung base. He is diagnosed with community acquired pneumonia and started on IV ampicillin and supplemental O2 via nasal cannula before being admitted to the medical-surgery unit for further care.

**I-PASS Rounds Role Play Simulation Activity**

**Goal:** To familiarize team members with the varying roles in PFC-IPASS.

**You are part of the rounds team in the role of the faculty member/attending**

Please review the case information and your key behaviors as listed below (don’t overact but embrace your role)

- You realize that the intern is not looking at the family and need to redirect
- You are a little concerned with the amount of medical jargon in the presentation and need to try to redirect the intern
- You are wondering if the family is comfortable synthesizing

**Case scenario:** Max is a 10-year-old male with known history of asthma, who was brought to the Emergency Department, accompanied by his mother, Mrs. Carter, with a 4-day history of worsening cough, and fever Tmax 102°F. Despite at home treatments with albuterol, Max developed tachypnea and labored breathing. He also has had decreased oral intake and only 3 urinary voids in the last 24 hours. On exam, Max has a temperature of 101.7°F, HR of 110 bpm, RR of 32 breaths/min, and pulse oximetry of 90% in room air. He is noted to have crackles and decreased aeration over the left lung base. He is diagnosed with community acquired pneumonia and started on IV ampicillin and supplemental O2 via nasal cannula before being admitted to the medical-surgery unit for further care.

**I-PASS Rounds Role Play Simulation Activity**

**Goal:** To familiarize team members with the varying roles in PFC-IPASS.

**You are part of the rounds team in the role of the nurse**

Please review the case information and your key behaviors as listed below (don’t overact but embrace your role)

- You know you are supposed to share the overnight events after the introductions but don’t know what to do when the intern keeps talking
- You do share if the senior resident redirects the intern or if the intern gets up to the first problem without stopping

**Case scenario:** Max is a 10-year-old male with known history of asthma, who was brought to the Emergency Department, accompanied by his mother, Mrs. Carter, with a 4-day history of worsening cough, and fever Tmax 102°F. Despite at home treatments with albuterol, Max developed tachypnea and labored breathing. He also has had decreased oral intake and only 3 urinary voids in the last 24 hours. On exam, Max has a temperature of 101.7°F, HR of 110 bpm, RR of 32 breaths/min, and pulse oximetry of 90% in room air. He is noted to have crackles and decreased aeration over the left lung base. He is diagnosed with community acquired pneumonia and started on IV ampicillin and supplemental O2 via nasal cannula before being admitted to the medical-surgery unit for further care.

**I-PASS Rounds Role Play Simulation Activity**

**Goal:** To familiarize team members with the varying roles in PFC-IPASS.

**You are part of the rounds team in the role of the senior resident**

Please review the case information and your key behaviors as listed below (don’t overact but embrace your role)

- You notice your intern forgot to let the nurse speak second so you try to redirect
- You think the intern’s presentation has gone really well so you don’t need to address anything
- You are trying to make sure the other intern remembers to do the rounds report – you redirect if they are not doing it

**Case scenario:** Max is a 10-year-old male with known history of asthma, who was brought to the Emergency Department, accompanied by his mother, Mrs. Carter, with a 4-day history of worsening cough, and fever Tmax 102°F. Despite at home treatments with albuterol, Max developed tachypnea and labored breathing. He also has had decreased oral intake and only 3 urinary voids in the last 24 hours. On exam, Max has a temperature of 101.7°F, HR of 110 bpm, RR of 32 breaths/min, and pulse oximetry of 90% in room air. He is noted to have crackles and decreased aeration over the left lung base. He is diagnosed with community acquired pneumonia and started on IV ampicillin and supplemental O2 via nasal cannula before being admitted to the medical-surgery unit for further care.
