# Supplementary material for: Patient and Family-Centered I-PASS SCORE Program: Resident and Advanced Care Provider Training Materials
Source: MedEdPORTAL. 2022 Aug 9;18:11267. doi: 10.15766/mep_2374-8265.11267 (PMC9360201; doi:10.15766/mep_2374-8265.11267)
Supplement: Supplementary file 1 — PFC I-PASS Core Content Module.m4vHealth Literacy Exercise.docxCore Content Module Evaluation.docxResident and Advanced Care Provider Workshop.pptxResident and Advanced Care Provider Facilitator Guide.docxPFC I-PASS Structure Role-Play.docxPFC I-PASS Rounds Role-Play.docxWritten Handoff for Rounds Role-Play.docxObserver Tool for Rounds Role-Play.docxRounds Report Simulation Activity.docxWorkshop Evaluation.docxBad Example of PFCR.mp4Good Example of PFCR.mp4Tips for Conducting Virtual Teaching Sessions.docx [file mep_2374-8265.11267-s001.zip › H. Written Handoff for Rounds Role-Play.docx]

**Written Handoff For Rounds Role-Play**

|  | **I** | **P** | **A** | **S** | **S** |
| --- | --- | --- | --- | --- | --- |
|  | **Illness**  **Severity** | **Patient Summary** | **Action List** | **Situation Awareness &**  **Contingency Planning** | **Synthesis by**  **Receiver** |
| M10E **Carter, Max**  MRN: 24876401  Visit: 98203  Attending: Parker, Janet  Code Status: Full  Allergies: NKDA  Wt: 18 kg  Access: PIV  Resident: Rice, Anthony  **Medications**  Fluticasone  Albuterol Q2h  Solumedrol  Ampicillin | Watcher | 10 year old PMHx asthma now admitted with respiratory distress and likely community acquired pneumonia  Presents with 4 days of worsening cough, 2 days of fever to 102, 1 day of worsening tachypnea, decreased po intake and urine output. VS in ED significant for T101.7, P110, R30, Pox 90%RA, with crackles, decreased aeration L base occasional scattered wheeze given Ampicillin and a NS bolus and admitted to med-surg.  • Pneumonia – focality on exam at L base with hypoxia, on NC 2 lpm, Ampicillin  • Asthma – some scattered wheeze, on Albuterol Q2h and Solumedrol  • Dehydration – received bolus in ED, allowing po, IVF at 1xm | - Follow respiratory exam closely (every 2h for now) - Monitor fever curve - Monitor ins and outs | - If increased oxygen needs or tachypnea, then obtain CXR to evaluate for effusion - If decreased urine output or po then fluid bolus - If persistent fevers, then repeat CBC | SYNTHESIS BY RECEIVER |
