# Supplementary material for: Patient and Family-Centered I-PASS SCORE Program: Resident and Advanced Care Provider Training Materials
Source: MedEdPORTAL. 2022 Aug 9;18:11267. doi: 10.15766/mep_2374-8265.11267 (PMC9360201; doi:10.15766/mep_2374-8265.11267)
Supplement: Supplementary file 1 — PFC I-PASS Core Content Module.m4vHealth Literacy Exercise.docxCore Content Module Evaluation.docxResident and Advanced Care Provider Workshop.pptxResident and Advanced Care Provider Facilitator Guide.docxPFC I-PASS Structure Role-Play.docxPFC I-PASS Rounds Role-Play.docxWritten Handoff for Rounds Role-Play.docxObserver Tool for Rounds Role-Play.docxRounds Report Simulation Activity.docxWorkshop Evaluation.docxBad Example of PFCR.mp4Good Example of PFCR.mp4Tips for Conducting Virtual Teaching Sessions.docx [file mep_2374-8265.11267-s001.zip › Educational_Summary_Report_11267.pdf]

MedEdPORTAL® The AAMC Journal of Teaching and Learning Resources

Original Publication Open Access

Patient and Family-Centered I-PASS SCORE Program: Resident and Advanced Care Provider Training Materials

Kheyandra D. Lewis, MD, MEd*, Lauren Destino, MD, Jennifer Everhart, MD, Anupama Subramony, MD, MBA, Benard Dreyer, MD, Brenda Allair, MEd, Michele Anderson, MPH, CHES, Jennifer Baird, PhD, MPH, MSW, RN, Zia Bismilla, MD, MEd, Brian Good, MB, BCh, BAO, Jennifer Hepps, MD, Alisa Khan, MD, MPH, Nicholas Kuzma, MD, Christopher P. Landrigan, MD, MPH, Katherine Litterer, Theodore C. Sectish, MD, Nancy D. Spector, MD, H. Shonna Yin, MD, MS, Clifton E. Yu, MD, Sharon Calaman, MD, Jennifer K. O'Toole, MD, MEd

*Corresponding author: KDL62@drexel.edu

Abstract

Introduction: Patient and family-centered rounds (PFCRs) are an important element of family-centered care often used in the inpatient pediatric setting. However, techniques and best practices vary, and faculty, trainees, nurses, and advanced care providers may not receive formal education in strategies that specifically enhance communication on PFCRs. **Methods:** Harnessing the use of structured communication, we developed the Patient and Family-Centered I-PASS Safer Communication on Rounds Every Time (SCORE) Program. The program uses a standardized framework for rounds communication via the I-PASS mnemonic, principles of health literacy, and techniques for patient/family engagement and bidirectional communication. The resident and advanced care provider training materials, a component of the larger SCORE Program, incorporate a flipped classroom approach as well as interactive exercises, simulations, and virtual learning options to optimize learning and retention via a 90-minute workshop. **Results:** Two hundred forty-six residents completed the training and were evaluated on their knowledge and confidence regarding key elements of the curriculum. Eighty-eight percent of residents agreed/strongly agreed that after training they could activate and engage families and all members of the interprofessional team to create a shared mental model; 90% agreed/strongly agreed that they could discuss the roles/responsibilities of various team members during PFCRs. **Discussion:** The Patient and Family-Centered I-PASS SCORE Program provides a structured framework for teaching advanced communication techniques that can improve provider knowledge of and confidence with engaging and communicating with patients/families and other members of the interprofessional team during PFCRs.

Keywords

Family-Centered Rounds, Communication Skills, Health Literacy, Hospital Medicine, Interprofessional Education, Pediatrics, Quality Improvement/Patient Safety

Educational Objectives

By the end of this activity, learners will be able to:

1. Activate and engage patients, families, nurses, and additional members of the interprofessional team to create a shared mental model using the Patient and Family-Centered I-PASS Rounds Do Every Time Process.
2. Apply principles of health literacy such as plain language and teach-back, as well as techniques for bidirectional communication, to promote discussion.
3. Incorporate I-PASS structured communication as an organizing framework for patient and family-centered rounds.
4. Discuss the roles and responsibilities of various team members during patient and family-centered rounds.
5. Demonstrate effective use of written information to facilitate communication with patients, families, and the interprofessional team.
6. List appropriate educational activities for patient and family-centered rounds that are tailored to patient and family needs/preferences.

Citation:
Lewis KD, Destino L, Everhart J, et al. Patient and Family-Centered I-PASS SCORE Program: resident and advanced care provider training materials. *MedEdPORTAL.* 2022;18:11267.
https://doi.org/10.15766/mep_2374-8265.11267

Introduction

Patient and family-centered rounds (PFCRs) have become the preferred method of conducting rounds in pediatric inpatient

Copyright © 2022 Lewis et al. This is an open-access publication distributed under the terms of the Creative Commons Attribution license.

medicine.[1] PFCRs provide an ideal opportunity for health care providers, patients, and families to engage in shared decision-making. The American Academy of Pediatrics and the Agency for Healthcare Research and Quality have called for PFCRs as an approach to improve patient engagement, and PFCRs have become an important element of delivering patient and family-centered care.[2,3] The positive impact of this rounding approach has been detailed throughout the literature.[1] However, there is variability across institutions and specialties in how PFCRs are taught and conducted with respect to location, participants, and the role of participants on rounds.[1]

Often, the primary presenters for PFCRs are resident physicians. However, residents may not have received dedicated education on how to present during PFCRs, and those who have received training often feel that it was not of high quality.[1] Many skills are needed to conduct PFCRs well, including general bedside communication etiquette such as introductions and positioning, use of lay language, relationship building, and demonstration of respect for the patient and family.[4] Interns in particular may have insufficient training in a number of these skills as exposure to PFCRs in medical school is variable depending on the institution and clinical rotations.[5-7] To address this variability and to optimize the opportunity to enhance communication and engage patients, families, and other members of the interprofessional team, the Patient and Family-Centered (PFC) I-PASS Program was developed.

In 2014, the PFC I-PASS Program, an evidence-based, standardized communication intervention coproduced by families, nurses, and physicians, was part of the intervention in the PFC I-PASS Study, which explored how enhancing communication utilizing a structured framework for rounds, principles of health literacy, and techniques for family engagement and bidirectional communication could improve patient safety. The program, developed utilizing an iterative approach, comprises a high-reliability framework for rounds communication anchored by the I-PASS mnemonic (I: illness severity, P: patient summary, A: action list, S: situational awareness and contingency planning, and S: synthesis by receiver), a written summary to complement information exchanged on rounds in real time, and training in structured interprofessional communication. All these components harness core principles of health literacy. The implementation of the program at seven pediatric institutions in North America was associated with a 38% reduction in harmful medical errors and improved patient and family experience without prolonging the duration of rounds or decreasing teaching on rounds.[8]

Leveraging the success of the original PFC I-PASS Rounds Program, the PFC I-PASS Safer Communication on Rounds Every Time (SCORE) Program spread the educational bundle to 21 sites across North America as part of the Society of Hospital Medicine Mentored Implementation Program, which pairs sites with experienced mentors to enact improvement initiatives.[9] Our study group has been successful in the creation of programming through mentored implementation, as demonstrated by our previous work with provider handoffs, which has also been published in *MedEdPORTAL*.[10-17] To develop the PFC I-PASS SCORE Program, we took lessons learned from implementation of the original PFC I-PASS Program and made adaptations and refinements to create a more effective program for the SCORE project. The PFC I-PASS SCORE Program consists of three core intervention elements: (1) structured verbal communication on rounds, (2) structured written communication on rounds, and (3) advanced techniques for communication, teamwork, and application of health literacy best practices to establish a shared mental model among all members of the care team, including the patient and family. The revised curriculum includes a flipped classroom[18] approach, enhanced strategies to engage learners and improve their knowledge retention, identification of defined roles and speaking processes for rounds, and options to allow for virtual or modified in-person options in response to the COVID-19 pandemic.

The PFC I-PASS SCORE resident and advanced care provider training materials include all self-study, in-person, and virtual learning materials needed to effectively train residents and advanced care providers in the program. While the resident and advanced care provider training materials serve as an independent resource for training these groups of individuals, we recommend using them along with the other complementary materials from the PFC I-PASS SCORE Program. The entire PFC I-PASS SCORE Program is a multifaceted collection of modules that provides training and strategic plans for leading every aspect of culture change required to put an effective rounding bundle in place that engages patients, families, and all members of the interprofessional team. The other modules focus on medical students, faculty members, and how to support the overall implementation using best practices in quality improvement and human factors research. While the PFC I-PASS SCORE Program included mentorship from external experts for each site, we believe we have compiled all of the critical training materials and best practices for implementation and sustainment as part of the entire package without need for an external mentor. This suite of materials will allow anyone to successfully implement this program at their home institution.

Copyright © 2022 Lewis et al. This is an open-access publication distributed under the terms of the Creative Commons Attribution license.

MedEdPORTAL features a number of publications on PFCRs.[19-22] These existing educational modules specifically address teaching medical students, promoting autonomy of learners, tips for oral presentations, and assessing and providing feedback to learners on PFCRs. Our module adds to these resources by specifically focusing on teaching residents and including important techniques for PFCRs that can promote general communication, the interprofessional care team, shared understanding, and, importantly, patient safety.

Methods

This project was granted expedited Institutional Review Board (IRB) approval from Boston Children's Hospital. Participating sites secured local IRB approval as determined by their individual institutions.

Development of the Curriculum

The original PFC I-PASS training materials were developed for the PFC I-PASS Study that occurred from 2014 to 2016.[8] These materials were coproduced in a rigorous fashion with input from medical educators, nurses, health literacy experts, health services researchers, patient safety experts, and, most importantly, patients and family members. The materials took 1 year to develop using Kern's six steps for curriculum development[23] and a conceptual model for PFCRs. For the PFC I-PASS SCORE Program, the materials underwent full revision and editing based upon feedback from the original study sites and subjects. Figure 1 details the entire evolution of the PFC I-PASS SCORE Program. The program consisted of self-study training components (Appendices A-C) and in-person training components (Appendices D-N). Highlights of the edits that were made to the training materials include the following:

- To shorten the length of the in-person training workshops, which were upwards of 3 hours in the first PFC I-PASS Study, we incorporated a flipped classroom[18] approach by developing a 29-minute core content module (Appendix A) and an approximately 10-minute health literacy exercise (Appendix B) for residents or advanced care providers to complete in advance of the in-person training. This helped to decrease the cognitive load placed on learners and to reinforce key concepts. The new in-person workshop (Appendix D) took roughly 90 minutes to complete in total.
- In response to the COVID-19 pandemic, which limited in-person training options, we created workshops (Appendix D), simulations (Appendices F and G), and activities (Appendix J) that could be done on a virtual training platform. This provided flexibility for training options based upon space and personal protective equipment limitations.
- To address feedback that individual learner types were unsure of their roles on rounds, we created a Do Every Time Process (Figure 2) detailing the exact order of rounds flow and defining clear roles for each team member before and during rounds.

Overview of the Curricular Materials

Self-study training components: The self-study training components (Appendices A-C) are intended to be completed prior to the in-person training. They provide background information on PFCRs, the history of PFC I-PASS, the importance of communication and health literacy, and how to incorporate the PFC I-PASS format, as well as an opportunity to develop a written update utilizing health literacy principles. The self-study training components should take approximately 45 minutes for learners to complete and include the following:

- The PFC I-PASS core content module (Appendix A), a 29-minute narrated PowerPoint video.
- The 10-minute health literacy exercise (Appendix B) in which learners are provided with a detailed patient admission prompt and are instructed to develop a written update.
- The 5-minute core content module evaluation (Appendix C).

In-person or synchronous virtual workshop components: The following educational materials, accompanied by a facilitator guide (Appendix E), are intended to be used during the 90-minute workshop (Appendix D). This workshop (Appendix D) reinforces the key concepts from the self-study training (Appendices A-C), allowing discussion of the health literacy exercise (Appendix B) and practice through interactive exercises of the structured communication utilized in the PFC I-PASS rounding format (Appendix F). Through use of a PowerPoint presentation and additional interactive exercises (Appendix D), residents and advanced care providers are oriented to the varying roles and responsibilities of all members of the interprofessional team on PFCRs, special considerations for situations that may impact rounds communication, and advanced communication techniques and considerations based on developmental age, limited English proficiency, discussion of sensitive topics, and teaching on rounds. The in-person training components consist of the following:

- The resident/advanced care provider workshop (Appendix D), a 90-minute PowerPoint presentation for use in person or virtually in a synchronous session.
- The resident/advanced care provider workshop facilitator guide (Appendix E), which provides facilitators with

Copyright © 2022 Lewis et al. This is an open-access publication distributed under the terms of the Creative Commons Attribution license.

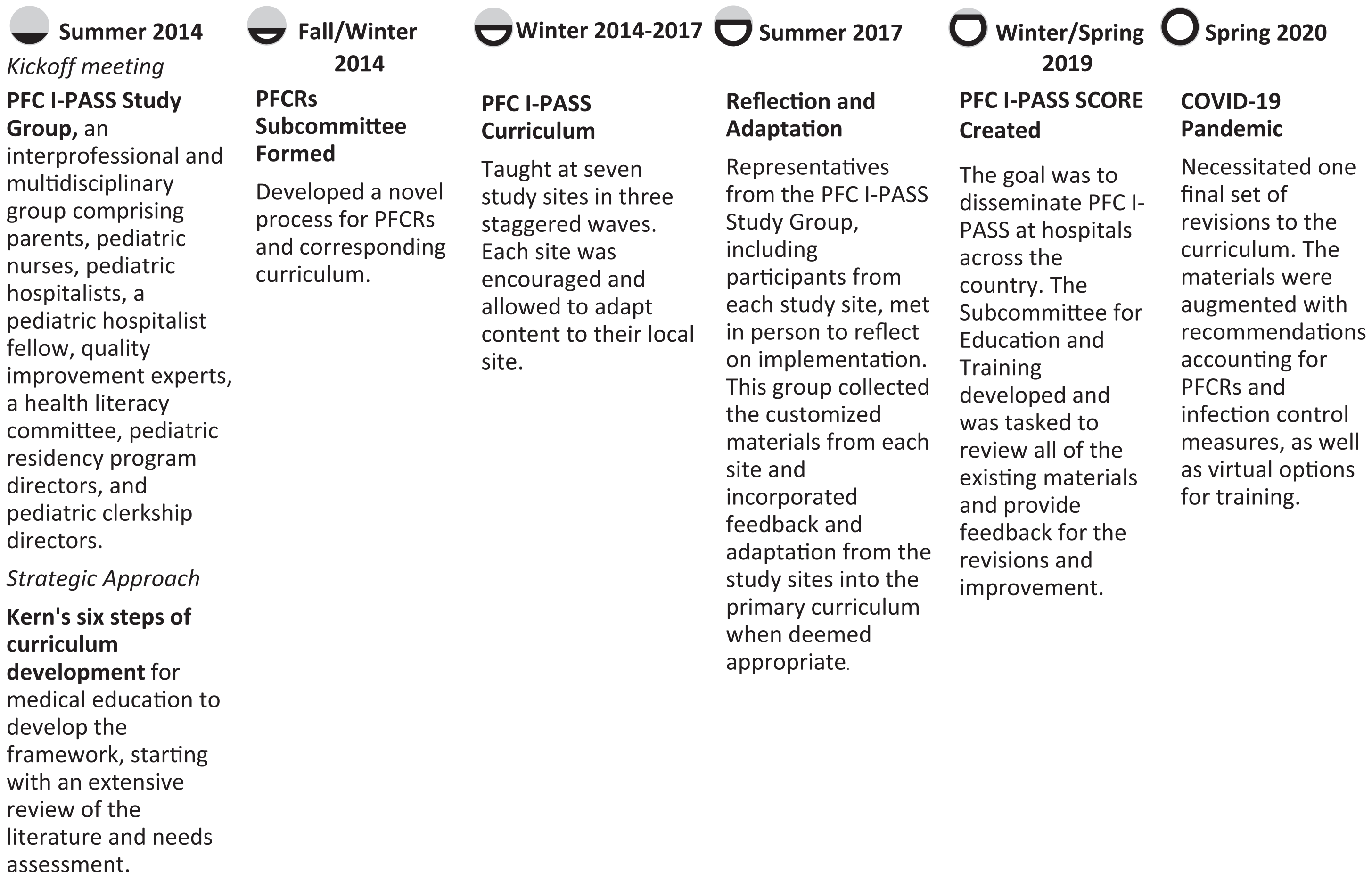

Figure 1. Evolution of the PFC I-PASS SCORE Program. Abbreviations: PFC, patient and family-centered; PFCRs, patient and family-centered rounds; SCORE, safer communication on rounds every time.

recommendations and guidance for each accompanying activity within the workshop.

- The PFC I-PASS structure role-play materials, a 30-minute interactive role-play exercise with participants 1-4 (Appendix F).
- The PFC I-PASS rounds role-play materials with the roles of presenting intern, nonpresenting intern, parent, patient, faculty member, nurse, and senior resident (Appendix G); the written handoff for the rounds role-play (Appendix H); and the observer tool for the rounds role-play (Appendix I): This role-play exercise is approximately 15 minutes inclusive of discussion.
- The rounds report simulation activity (Appendix J), a 5-minute written exercise.
- The workshop evaluation (Appendix K).
- Video example of a bad PFCR (Appendix L): just over 4 minutes.
- Optional video: example of a good PFCR (Appendix M): just under 8 minutes
- Tips for conducting virtual teaching sessions (Appendix N).

Implementation of Curriculum

The PFC I-PASS SCORE resident and advanced care provider training materials incorporate a flipped classroom[18] approach, requiring residents and/or advanced care providers to complete self-study training components prior to attending in-person training. For the initial rollout of the program, training of residents and advanced care providers should take place approximately 1 or 2 months before. Future refreshers can take place closer to the learners' start date, for example, during an orientation. Key planning steps include the following:

1. Learners should review the PFC I-PASS core content module (Appendix A) and complete the health literacy

Copyright © 2022 Lewis et al. This is an open-access publication distributed under the terms of the Creative Commons Attribution license.

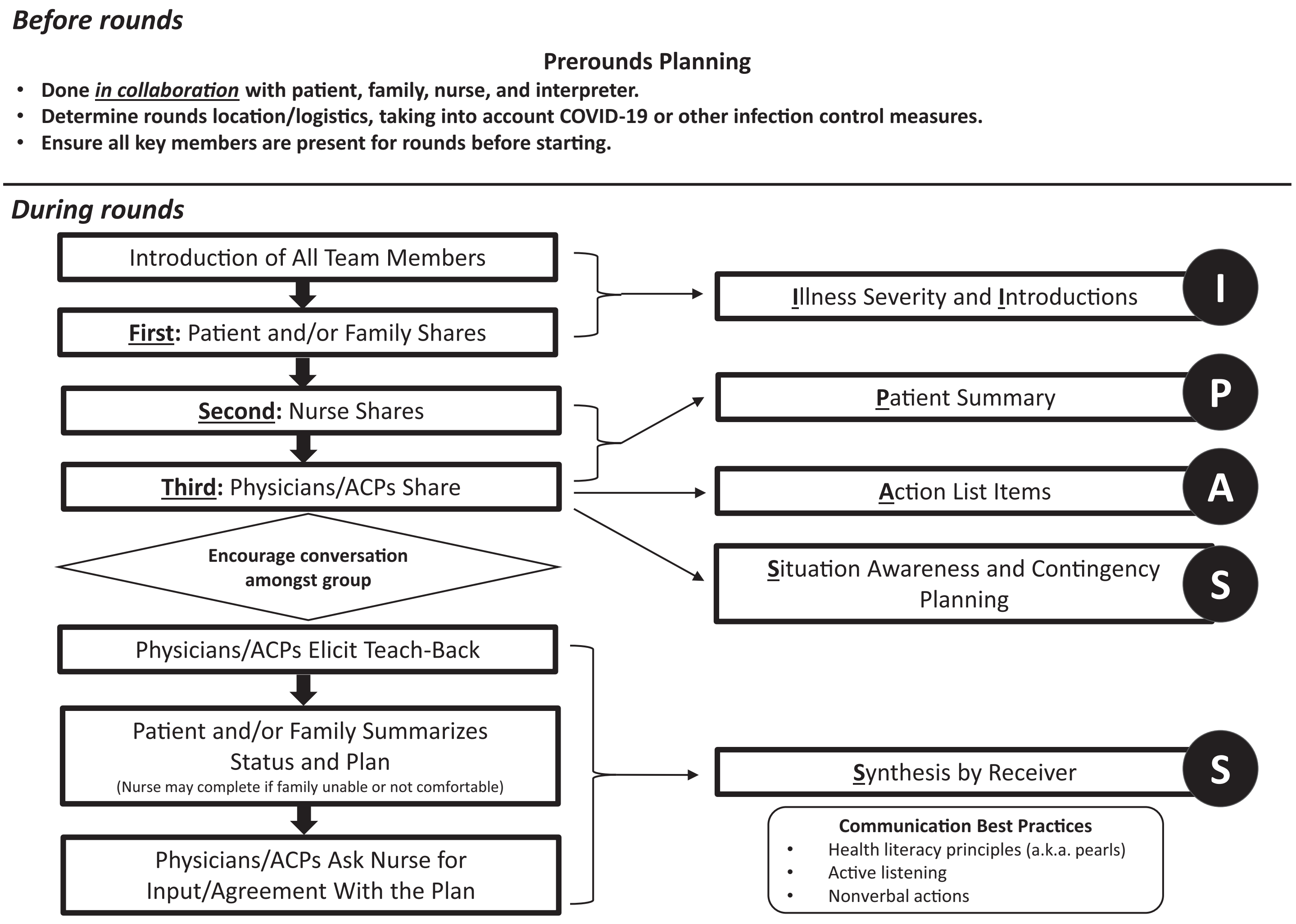

Figure 2. Patient and Family-Centered I-PASS Rounds: Do Every Time Process. Abbreviation: ACP, advanced care provider.

exercise (Appendix B) at least 2-4 weeks prior to the in-person training.

2. Utilize a space where learners can easily move around into small groups, if the session is being conducted in person. The room will need audiovisual capabilities for projecting PowerPoint slides and showing video elements. Some options for scheduling training include resident orientation, educational retreats, or conferences.
3. Training should be led by faculty who have familiarity with the curricular components. When available, the addition of patient/family advisors and nursing facilitators can add amplified perspectives to the delivery of the educational content.
4. For the PFC I-PASS structure role-play (Appendix F), there should be a ratio of one faculty to four learners. The faculty can attend during the role-play and step away for other portions of the workshop to facilitate scheduling. After the program is established, in future trainings senior residents or advanced care providers who have previously undergone training can serve in a faculty facilitator role for this activity.
5. For the PFC I-PASS rounds role-play (Appendix G), the role-play elements of the training materials are delineated based on year of training (i.e., intern or senior resident); however, role assignments can be adapted for other types of advanced care providers (i.e., primary presenter rather than intern).
6. When conducting the workshop virtually, within the slide deck provided for the resident/advanced care provider workshop (Appendix D) are embedded prompts that recommend content to share in person versus virtually. This requires a facilitator to edit or hide specific slides prior

Copyright © 2022 Lewis et al. This is an open-access publication distributed under the terms of the Creative Commons Attribution license.

to the session. Further guidance on how to conduct the workshop virtually can be found in the tips for conducting virtual teaching sessions (Appendix N).

7. Handouts for the PFC I-PASS structure role-play (Appendix F), PFC I-PASS rounds role-play (Appendices G-I), rounds report simulation activity (Appendix J), and workshop evaluation (Appendix K) should be printed in advance of the workshop, if it is being led in person, or distributed electronically for the virtual session. It is also helpful to distribute the resident/advanced care provider workshop facilitator guide (Appendix E) to the facilitators in ahead of time.

During the 90-minute resident/advanced care provider workshop (Appendix A), learners participate in a variety of highly interactive exercises and hands-on activities to build knowledge and promote retention.

1. First, the workshop facilitator frames the objectives and agenda for the session. This includes showing an approximately 4-minute video (Appendix L) illustrating what could happen if providers do not engage the patient/family in PFCRs.
2. Next, learners review key principles of health literacy by sharing their completed health literacy exercise (Appendix B) with a partner for 5 minutes.
3. After reviewing the health literacy exercise (Appendix B), the facilitator spends 5-7 minutes discussing concepts of standard communication techniques to establish a shared mental model using the Do Every Time Process as well the structural aspects of the PFC I-PASS rounding format.
4. Learners break into groups of four for the PFC I-PASS structure role-play (Appendix F) for the next 30 minutes (15 minutes per case). Each learner has the opportunity to play the role of the primary presenter, a senior resident, or one of two observers for two distinct cases. Each participant is provided with a case vignette and accompanying medical information, as well as instructions for their role. Each group of four learners should have a faculty facilitator to answer specific questions and facilitate ongoing feedback.
5. Next, through the workshop PowerPoint presentation (Appendix D), the facilitator helps learners orient to the varying roles and responsibilities of all members of the interprofessional team on PFCRs. Learners are introduced to and discuss special considerations for situations that can impact rounds communication, including nonverbal communication and team member positioning in room, utilization of computers during rounds, adherence to infection prevention precautions, and protective personal equipment, as well as advanced communication techniques and considerations based on developmental age, limited English proficiency, and discussion of sensitive topics. This section ends with a 10-minute large-group discussion exploring perceived benefits of and participant concerns about the role assignments.
6. Learners then engage in the PFC I-PASS rounds role-play (Appendix G). This activity takes approximately 10 minutes and requires seven participants to volunteer to act in the scripted roles portrayed in Appendix G. Additionally, the participants acting in the role-play need the written handoff for the rounds role-play (Appendix H). The instructions for the roles involve some challenging rounding scenarios, such as the primary presenter utilizing medical jargon. The remaining learners serve in the role of observers, utilizing the observer tool for the rounds role-play (Appendix I), and participate in a large-group discussion exploring aspects that went well, opportunities for improvement, and how they might have handled the situation encountered (e.g., needing to redirect the primary presenter).
7. Through the PowerPoint presentation (Appendix D), learners are oriented to the rounds report, a written document that augments verbal communication on rounds and summarizes key points from rounds for the patient/family, followed by an opportunity to practice the completion of a rounds report (Appendix J). The completion of the rounds report activity should take about 5 minutes.
8. The workshop concludes with recommendations on how to incorporate teaching points that can be optimized at the bedside for all members of the team and how to identify what topics and focus are more fitting in another educational environment.

Evaluation of the Curriculum

The PFC I-PASS SCORE resident physician/advanced care provider training was evaluated using both process and outcome measures via two separate postparticipation surveys (Appendices C and K) corresponding to the self-study and in-person training components, respectively. Process measures included assessment of training penetration (number of training sessions at each site and number of residents/advanced care providers trained at each site). Additionally, surveys collected demographic data, including provider type, year in training,

Copyright © 2022 Lewis et al. This is an open-access publication distributed under the terms of the Creative Commons Attribution license.

gender, age, and race and ethnicity. Outcome measures included asking learners to self-rate their ability to activate and engage families and interprofessional team members on rounds, apply health literacy principles, incorporate I-PASS structured communication on PFCRs, demonstrate use of written communication to effectively facilitate communication with families and the interprofessional team, list appropriate educational activities for PFCRs, and discuss roles and responsibility for various team members. Participants were also asked about the degree to which the workshop's materials provided knowledge and skills relevant to their patient care activities, the balance between didactic and interactive elements, and the pace and length of the workshop.

Results

A total of 246 residents underwent the PFC I-PASS SCORE resident physician/advanced care provider training from August 2019 through August 2020. Of the 246 residents who participated, 135 were interns (55%), 61 were second-year residents (25%), and 50 were third-year residents (20%). No advanced care providers completed the self-study training components or participated in the in-person training.

Self-Study Training Components

Overall, 59% of residents completed the self-study training components inclusive of the PFC I-PASS core content module (Appendix A), the health literacy exercise (Appendix B), and the core content module evaluation (Appendix C) prior to attending the in-person training. Of those residents who completed the self-study components, most responded to the open-ended prompt "List the most effective elements of the core content module" with the example video embedded within. Additional responses included becoming familiar with the I-PASS framework and the ability to learn at one's own pace.

In-Person Training Components

Measures were based on resident self-rating utilizing a 5-point scale (*strongly disagree, disagree, neutral, agree, strongly agree*). Residents were asked to delineate their perceived knowledge and skill ability regarding the following objectives after completion of the training:

1. Activate and engage families and all members of the interprofessional team to create a shared mental model.
2. Apply health literacy principles to improve communication.
3. Incorporate I-PASS structured communication as an organizing framework for family-centered rounds.
4. Demonstrate effective use of written communication to facilitate communication with families and the interprofessional team.
5. Discuss the roles and responsibilities of various team members during PFCRs.

Most residents agreed or strongly agreed that that the training provided knowledge and confidence in skills ability for all five stated objectives, with objective 1 (Activate and engage families and all members of the interprofessional team to create a shared mental model) and objective 5 (Discuss the roles and responsibilities for various team members during PFCRs) rated the highest, at 88% and 90%, respectively (Table 1).

Additionally, residents were asked to rate the training provided in the workshop using the same 5-point scale for the following outcome measures:

1. Provided me with knowledge and skills relevant to my patient care activities.
2. Was designed with an appropriate balance of didactic and interactive elements.
3. Had an appropriate pace.
4. Seemed to be the correct length to address the content.

Overall, most respondents rated the training highly, agreeing or strongly agreeing regarding provision of knowledge and skills relevant to patient care activities (82% of respondents) and the design of the training balancing didactic and interactive elements (82% of respondents). Although 80% of respondents found

Table 1. Resident Attitudes Regarding Whether Training Participation Provided Knowledge and Skills Ability to Perform the Following Activities on Patient and Family-Centered Rounds

Activity	Respondents Replying *Agree* or *Strongly Agree* No.	%
After participating in the training, I am able to:		
Activate and engage families and all members of the interprofessional team to create a shared mental model.	216	88
Apply health literacy principles to improve communication.	209	85
Incorporate I-PASS structured communication as an organizing framework for family-centered rounds.	200	81
Demonstrate effective use of written communication to facilitate communication with families and the interprofessional team.	203	83
Discuss the roles and responsibilities for various team members during patient and family-centered rounds.	221	90

Copyright © 2022 Lewis et al. This is an open-access publication distributed under the terms of the Creative Commons Attribution license.

the training to have an appropriate pace, only 75% found the length to be correct to address the content (Table 2). In open-prompt responses, some participants suggested that the didactic portions in the in-person training be further trimmed down as they found it repetitive, and many would have liked to dedicate more time to the interactive exercise components.

Discussion

Participation in the PFC I-PASS SCORE resident physician/advanced care provider training improved residents' knowledge and increased their confidence in their ability to effectively communicate with patients, families, and other members of the interprofessional team during PFCRs. Utilizing multiple interactive techniques to solidify new concepts provided residents with self-reported confidence in engaging and activating families using communication strategies to achieve a shared mental model regarding patient care and application of health literacy principles. Additionally, residents reported confidence in their abilities to use the I-PASS structured communication tool to communicate with families and members of the interprofessional team. Using Kirkpatrick's model,[24] this curriculum was well received (Level 1) and improved knowledge and confidence (Level 2).

Development of the PFC I-PASS SCORE Program involved experts in a variety of fields, including family-centered care, medical education, health literacy, and quality improvement. This expertise added to the robustness, quality, and uniqueness of the curriculum. The use of a flipped classroom[18] approach supplemented by simulations and interactive exercises enhanced the applicability of the tools in real-life settings. The balance of didactic and interactive elements was well received by learners. At the onset of the COVID-19 pandemic, adaptations for curricular training were made to accommodate the need for virtual training platforms. Furthermore, materials were augmented with recommendations on how to conduct PFCRs while maintaining infection control procedures and safety measures.

Table 2. Resident Feedback on the Training

Feedback	Respondents Replying *Agree* or *Strongly Agree* No.	%
This workshop:		
Provided me with knowledge and skills relevant to my patient care activities.	203	82
Was designed with an appropriate balance of didactic and interactive elements.	203	82
Had an appropriate pace.	197	80
Seemed to be the correct length to address the content.	184	75

A novel strength of this program is the role of nonphysicians in the coproduction of the training. As a best practice, we recommend inviting trained nurses, patient/family advisors, and/or additional ancillary staff within one's own institution to be facilitators who can provide additional perspective and suggestions to enhance interprofessional collaboration. Training programs can also consider the use of resident champions, allowing more senior residents the opportunity to cofacilitate subsequent trainings or refreshers for new learners.

A limitation of this program includes the fact that although the curriculum focused on interprofessional communication, many of the workshops were not consistently attended or facilitated by interprofessional teams. Additionally, given variability from site to site, tailoring of curriculum content to fit the local context was necessary. A little over half of participating residents completed the self-study training materials. To further improve adherence, faculty may want to provide timely reminders and engage residency leadership to incorporate the completion of these activities during a time when residents are not challenged by competing tasks. Despite our attempts to decrease the overall duration of the training, many residents still found it to be lengthy. Facilitators can consider utilizing just-in-time training before beginning an inpatient rotation or other educational venues to reinforce skills or review concepts. To sustain culture change, we recommend ongoing provision of feedback and periodic faculty observations on rounds.

In summary, the PFC I-PASS SCORE resident physician/advanced care provider training improved resident knowledge and confidence globally in several aspects of PFC care, especially how to activate and engage families and all members of the interprofessional team to create a shared mental model utilizing a structured framework to conduct PFCRs with incorporation of key health literacy principles and enhanced communication techniques.

Appendices

A. PFC I-PASS Core Content Module.m4v

B. Health Literacy Exercise.docx

C. Core Content Module Evaluation.docx

D. Resident and Advanced Care Provider Workshop.pptx

E. Resident and Advanced Care Provider Facilitator Guide.docx

F. PFC I-PASS Structure Role-Play.docx

Copyright © 2022 Lewis et al. This is an open-access publication distributed under the terms of the Creative Commons Attribution license.

G. PFC I-PASS Rounds Role-Play.docx

H. Written Handoff for Rounds Role-Play.docx

I. Observer Tool for Rounds Role-Play.docx

J. Rounds Report Simulation Activity.docx

K. Workshop Evaluation.docx

L. Bad Example of PFCR.mp4

M. Good Example of PFCR.mp4

N. Tips for Conducting Virtual Teaching Sessions.docx

All appendices are peer reviewed as integral parts of the Original Publication.

Kheyandra D. Lewis, MD, MEd: Assistant Professor, Department of Pediatrics, Drexel University College of Medicine and St. Christopher's Hospital for Children; ORCID: https://orcid.org/0000-0001-7610-0487

Lauren Destino, MD: Clinical Associate Professor of Pediatrics, Department of Pediatrics, Stanford University School of Medicine and Lucile Packard Children's Hospital Stanford

Jennifer Everhart, MD: Clinical Associate Professor of Pediatrics, Department of Pediatrics, Stanford University School of Medicine and Lucile Packard Children's Hospital Stanford

Anupama Subramony, MD, MBA: Assistant Professor, Department of Pediatrics, Hofstra Northwell School of Medicine and Cohen Children's Medical Center

Benard Dreyer, MD: Professor, Department of Pediatrics, NYU Grossman School of Medicine and Hassenfeld Children's Hospital at NYU Langone

Brenda Allair, MEd: Family Mentor, Family Advisory Council, Boston Children's Hospital

Michele Anderson, MPH, CHES: Family Centered Care Parent Mentor, Family Centered Care Department, Lucile Packard Children's Hospital Stanford

Jennifer Baird, PhD, MPH, MSW, RN: Research Nurse Scientist, Institute for Nursing and Interprofessional Research, Children's Hospital Los Angeles

Zia Bismilla, MD, MEd: Associate Professor, Department of Pediatrics, University of Toronto and The Hospital for Sick Children

Brian Good, MB, BCh, BAO: Associate Professor, Department of Pediatrics, University of Utah and Primary Children's Hospital

Jennifer Hepps, MD: Assistant Professor, Uniformed Services University of the Health Sciences and Walter Reed National Military Medical Center

Alisa Khan, MD, MPH: Assistant Professor, Department of Pediatrics, Harvard Medical School and Boston Children's Hospital

Nicholas Kuzma, MD: Associate Professor, Department of Pediatrics, Drexel University College of Medicine and St. Christopher's Hospital for Children

Christopher P. Landrigan, MD, MPH: Professor, Department of Pediatrics, Boston Children's Hospital; Professor, Departments of Medicine and Neurology, Brigham and Women's Hospital and Harvard Medical School

Katherine Litterer: Family Mentor, Office of Experience, Boston Children's Hospital

Theodore C. Sectish, MD: Professor, Department of Pediatrics, Harvard Medical School and Boston Children's Hospital

Nancy D. Spector, MD: Professor, Department of Pediatrics, Drexel University College of Medicine

H. Shonna Yin, MD, MS: Associate Professor, Departments of Pediatrics and Population Health, NYU Grossman School of Medicine, NYC Health + Hospitals, and Bellevue Hospital Center

Clifton E. Yu, MD: Professor, Directorate for Education, Training, and Research, Uniformed Services University of the Health Sciences and Walter Reed National Military Medical Center

Sharon Calaman, MD: Professor, Department of Pediatrics, NYU Grossman School of Medicine and Hassenfeld Children's Hospital at NYU Langone

Jennifer K. O'Toole, MD, MEd: Professor, Departments of Pediatrics and Internal Medicine, University of Cincinnati College of Medicine and Cincinnati Children's Hospital Medical Center

---

Acknowledgments
We would like to acknowledge the members of the Patient and Family-Centered I-PASS SCORE Program, including the Coordinating Council, Education and Training Committee, Simulation Subcommittee, Implementation Committee, Scientific Oversight Committee, Scholarship Oversight Committee, Family Advisory Council, Nursing Advisory Council, Mentorship Committee, Physician Mentors, Family Mentors, and Nurse Mentors, as well as the Patient and Family-Centered I-PASS Education Executive Committee.

Disclosures
Drs. Calaman, Kuzma, Landrigan, O'Toole, and Spector have served as consultants for the I-PASS Patient Safety Institute.

Funding/Support
Dr. Christopher P. Landrigan received funding from the Patient-Centered Outcomes Research Institute (DI-2017C3-9232 and CDR-1306-03556).

Prior Presentations
Lewis K, Everhart J, Anderson M, et al. Do we really need another family-centered rounds workshop? Workshop presented virtually at: Association of Pediatric Program Directors Conference; March 26, 2021.

Lewis K, Everhart J, Baird J, et al. Really, another family-centered rounds workshop? Workshop presented virtually at: Pediatric Hospital Medicine Virtual Conference; August 6, 2021.

Ethical Approval
The Boston Children's Hospital Institutional Review Board approved this project.

Copyright © 2022 Lewis et al. This is an open-access publication distributed under the terms of the Creative Commons Attribution license.

References

1. Mittal V. Family-centered rounds. *Pediatr Clin North Am*. 2014;61(4):663-670. https://doi.org/10.1016/j.pcl.2014.04.003
2. Committee on Hospital Care; Institute for Patient- and Family-Centered Care. Patient- and family-centered care and the pediatrician's role. *Pediatrics*. 2012;129(2):394-404. https://doi.org/10.1542/peds.2011-3084
3. Family-Centered Rounds Toolkit. Agency for Healthcare Research and Quality. December 2017. Accessed June 13, 2022. https://www.ahrq.gov/patient-safety/resources/engage-tool/index.html
4. Muething SE, Kotagal UR, Schoettker PJ, del Rey JG, DeWitt TG. Family-centered bedside rounds: a new approach to patient care and teaching. *Pediatrics*. 2007;119(4):829-832. https://doi.org/10.1542/peds.2006-2528
5. Mittal VS, Sigrest T, Ottolini MC, et al. Family-centered rounds on pediatric wards: a PRIS network survey of US and Canadian hospitalists. *Pediatrics*. 2010;126(1):37-43. https://doi.org/10.1542/peds.2009-2364
6. Young HN, Schumacher JB, Moreno MA, et al. Medical student self-efficacy with family-centered care during bedside rounds. *Acad Med*. 2012;87(6):767-775. https://doi.org/10.1097/ACM.0b013e318253dcdb
7. Block L, Hutzler L, Habicht R, et al. Do internal medicine interns practice etiquette-based communication? A critical look at the inpatient encounter. *J Hosp Med*. 2013;8(11):631-634. https://doi.org/10.1002/jhm.2092
8. Khan A, Spector ND, Baird JD, et al. Patient safety after implementation of a coproduced family centered communication programme: multicenter before and after intervention study. *BMJ*. 2018;363(8179):k4764. https://doi.org/10.1136/bmj.k4764
9. Maynard GA, Budnitz TL, Nickel WK, et al. Mentored implementation: building leaders and achieving results through a collaborative improvement model. *Jt Comm J Qual Patient Saf*. 2012;38(7):301-310, AP1-AP3. https://doi.org/10.1016/s1553-7250(12)38040-9
10. Spector ND, Starmer AJ, Allen AD, et al.; I-PASS Education Executive Committee. I-PASS Handoff Curriculum: core resident workshop. *MedEdPORTAL*. 2013;9:9311. https://doi.org/10.15766/mep_2374-8265.9311
11. Rosenbluth G, Patel SJ, Destino LA, et al.; I-PASS Education Executive Committee. I-PASS Handoff Curriculum: campaign toolkit. *MedEdPORTAL*. 2013;9:9397. https://doi.org/10.15766/mep_2374-8265.9397
12. Calaman S, Hepps J, Spector ND, et al.; I-PASS Education Executive Committee. I-PASS Handoff Curriculum: handoff simulation exercises. *MedEdPORTAL*. 2013;9:9402. https://doi.org/10.15766/mep_2374-8265.9402
13. O'Toole J, Sectish T, Starmer AJ, et al.; I-PASS Education Executive Committee. I-PASS Handoff Curriculum: faculty development resources. *MedEdPORTAL*. 2013;9:9540. https://doi.org/10.15766/mep_2374-8265.9540
14. Starmer AJ, Landrigan C, Srivastava R, et al.; I-PASS Education Executive Committee. I-PASS Handoff Curriculum: faculty observation tools. *MedEdPORTAL*. 2013;9:9570. https://doi.org/10.15766/mep_2374-8265.9570
15. Calaman S, Spector ND, Starmer AJ, et al.; I-PASS Education Executive Committee. I-PASS Handoff Curriculum: computer module. *MedEdPORTAL*. 2013;9:9337. https://doi.org/10.15766/mep_2374-8265.9337
16. O'Toole J, Calaman S, Everhart J, et al. I-PASS Handoff Curriculum: medical student workshop. *MedEdPORTAL*. 2014;10:9854. https://doi.org/10.15766/mep_2374-8265.9854
17. O'Toole JK, Starmer AJ, Calaman S, et al.; I-PASS Study Education Executive Committee. I-PASS Mentored Implementation Handoff Curriculum: implementation guide and resources. *MedEdPORTAL*. 2018;14:10736. https://doi.org/10.15766/mep_2374-8265.10736
18. Hurtubise L, Hall E, Sheridan L, Han H. The flipped classroom in medical education: engaging students to build competency. *J Med Educ Curric Dev*. 2015;2:35-43. https://doi.org/10.4137/JMECD.S23895
19. Weisgerber M, Toth H, Brewer C, et al. The instructor's guide for the SOS-REACH (Suspected Observable Senior Resident Empowerment Action Checklist) and SREA-21: tools for evaluating senior resident empowerment during family-centered rounds. *MedEdPORTAL*. 2011;7:8547. https://doi.org/10.15766/mep_2374-8265.8547
20. Austin J, Bumsted T, Brands C. Teaching and evaluating oral presentations on family-centered rounds using the FREE TIPSS tool. *MedEdPORTAL*. 2013;9:9553. https://doi.org/10.15766/mep_2374-8265.9553
21. Zak S, Alcamo A, Herrmann L, et al. Medical student presentations on family-centered rounds: a workshop to teach an art form. *MedEdPORTAL*. 2016;12:10394. https://doi.org/10.15766/mep_2374-8265.10394
22. Vepraskas SH, Porada K, Hakjiev J, Lauck S, Toth H, Weisgerber M. Teaching inpatient bedside presenter empowerment actions during an interactive workshop. *MedEdPORTAL*. 2018;14:10702. https://doi.org/10.15766/mep_2374-8265.10702
23. Thomas PA, Kern DE, Hughes MT, Chen BY, eds. *Curriculum Development for Medical Education: A Six-Step Approach*. 3rd ed. Johns Hopkins University Press; 2016.
24. Kirkpatrick JD, Kirkpatrick WK. *Kirkpatrick's Four Levels of Training Evaluation*. ATD Press; 2016.

Received: November 26, 2021
Accepted: May 24, 2022
Published: August 9, 2022

Copyright © 2022 Lewis et al. This is an open-access publication distributed under the terms of the Creative Commons Attribution license.
